# Forest resilience under global environmental change: Do we have the information we need? A systematic review

**DOI:** 10.1371/journal.pone.0222207

**Published:** 2019-09-12

**Authors:** Inés Ibáñez, Kirk Acharya, Edith Juno, Christopher Karounos, Benjamin R. Lee, Caleb McCollum, Samuel Schaffer-Morrison, Jordon Tourville

**Affiliations:** School for Environment and Sustainability, University of Michigan, Ann Arbor, Michigan, United States of America; Chinese Academy of Forestry, CHINA

## Abstract

The capacity of forests to recover after disturbance, i.e., their resilience, determines their ability to persist and function over time. Many variables, natural and managerial, affect forest resilience. Thus, understanding their effects is critical for the development of sound forest conservation and management strategies, especially in the context of ongoing global environmental changes. We conducted a representative review, meta-analysis, of the forest literature in this topic (search terms “forest AND resilience”). We aimed to identify natural conditions that promote or jeopardize resilience, assess the efficacy of post-disturbance management practices on forest recovery, and evaluate forest resilience under current environmental changes.

We surveyed more than 2,500 articles and selected the 156 studies (724 observations) that compared and quantified forest recovery after disturbance under different contexts. Context of recovery included: resource gradients (moisture and fertility), post-disturbance biomass reduction treatments, species richness gradients, incidence of a second disturbance, and disturbance severity. Metrics of recovery varied from individual tree growth and reproduction, to population abundance, to species richness and cover. Analyses show management practices only favored recovery through increased reproduction (seed production) and abundance of recruitment stages. Higher moisture conditions favored recovery, particularly in dry temperate regions; and in boreal forests, this positive effect increased with regional humidity. Biomass reduction treatments were only effective in increasing resilience after a drought. Early recruiting plant stages benefited from increased severity, while disturbance severity was associated with lower recovery of remaining adult trees. This quantitative review provides insight into the natural conditions and management practices under which forest resilience is enhanced and highlights conditions that could jeopardize future resilience. We also identified important knowledge gaps, such as the role of diversity in determining forest resilience and the lack of data in many regions.

## Introduction

Forest ecosystems around the world are under the influence of numerous disturbances, including natural dynamics and anthropogenic actions. The persistence and functionality of these ecosystems is highly dependent on their capacity to recover, i.e., their resilience. Furthermore, under current global change, disturbance regimes and subsequent forest recovery will both be affected [1,2]. However, these changes are linked to a high degree of uncertainty about how forests will respond [3]. Under such circumstances, maintaining and promoting resilience may be the most optimal approach to cope with uncertainties associated with global change [4,5]. Thus, to be able to develop sound forest conservation and management plans we will need to better understand the mechanisms underlying forest resilience.

Resilience in ecological systems has been defined in multiple ways, e.g., resisting, absorbing, reorganizing, or transforming after stress [6,7,8]. In forests, the concept is mostly associated with recovery (i.e., performance after disturbance or “engineering resilience”), but resilience has also been characterized in terms of resistance (reduction in performance during disturbance, “ecological resilience”) [9,10], and resilience *per se* (post-disturbance performance with respect to pre-disturbance performance) [11,12]. For this study, we focused on plant performance as a metric of resilience since this is the most commonly reported measured of ecosystem recovery. We use the term recovery loosely as reported vegetation response metrics to a disturbance usually vary; these may include individual performance (tree growth or reproduction), population dynamics (abundance or canopy closure), or community dynamics (species diversity or biomass). The capacity of forests to recover with respect to these response metrics reflects their resilience or ability to ‘bounce back’ after stress.

What confers resilience to a forest will vary as a function of many intrinsic and extrinsic variables. Within a plant community, certain developmental stages can be more resilient to disturbance than others. For example, younger trees recover faster from drought than older individuals [13,14], but their mortality rates under stress can also be higher [15]. In some cases resilience will be linked to the recovery of individual species, while in others to the recovery of the whole community [16]. Furthermore, forests can be highly resilient to a disturbance they evolved with, e.g., fire-prone environments, but highly susceptible to new stressors, e.g., introduction of nuisance species [17], or to changes in the disturbance regime [18,19]. Diverse communities have higher chances of containing species that can contribute to recovery processes [20]; for example, higher tree species diversity mitigates defoliation in forests after drought [21]. The functional diversity associated with species richness also seems to be tightly related to resilience [22], e.g., in temperate and boreal forests hydraulic diversity increases forest resilience to drought [23].

Extrinsic variables, i.e., environmental context, can also affect resilience. Sites with high fertility and moisture levels have been linked to higher ecosystem resilience, as vegetation recovery is favored if resources are not limited [24,25]. Furthermore, resilience may also vary as a function of the frequency and severity of the disturbance [26,27]. Here, threshold dynamics may shape recovery if the vegetation responds by shifting towards a different stage [28,1].

Management practices affect resilience as well. Silvicultural systems vary widely, from single tree removal to clear cutting, resulting in very different recovery dynamics [29,30]. Pre- and post-disturbance biomass reduction treatments, implemented to improve tree growth, are common in both natural forests and plantations. Thinning in tree plantations ameliorates the negative effects of drought [31,32], as lower tree densities decrease competition for water. In Mediterranean ecosystems, prescribed fires are widely used to favor the regeneration of particular species [33]. And, fertilization practices, implemented in some commercial forests, can increase tree growth after disturbance [34,35].

Forests’ resilience will become even more relevant under the pressure of increasing disturbances associated with climate change [36,37,38]. Forecasted environmental changes will likely impact forest ecosystems, both through gradual changes in average climatic conditions and, most critically, by intensifying the frequency and magnitude of extreme weather events [39]. Drought and heat stress are already causing large scale tree mortality worldwide [40]. Fire regimes are shifting and becoming more severe due to both global warming and human management practices of forest ecosystems [41,42]. Furthermore, the negative impacts of human exploitation of these systems are exacerbated by other global change factors, including pollution and species introductions [43]. Since these trends are likely to continue, the best strategy to develop conservation and management plans that promote forest resilience is to integrate the fundamental mechanisms underlying forest responses to disturbance into these plans [16,44].

There is a vast body of literature on forest recovery after disturbance. Numerous reviews and meta-analyses have focused on either the development of broad resilience frameworks (i.e., qualitative reviews) or on resilience in specific biomes, or resilience after particular disturbances (i.e., meta-analyses) [16,45,46]. Leveraging on this work, we aimed at providing an intermediate level review on this topic, that is a review that identifies broad patterns but that is still backed by analysis of published data. For that we sampled a portion of the literature to carry out a broad evaluation of how natural factors and management practices may affect forest recovery. In particular, we were interested in assessing how context affected forest recovery after disturbance. Ultimately, our aim was to evaluate the information available and assess if this is sufficient to make inferences of forest resilience under the ongoing environmental changes forests are experiencing.

## Materials and methods

### Literature search and data extraction

Given the large body of work on forest recovery (>12,000 publications), we limited our search to studies that include the term ‘resilience’ (~2,500 publications). We are aware these only constitute a fraction of the literature published in this field, but this was our attempt to analyze an unbiased sample of the work published. On October 25^th^ 2017, we carried out a search in the Web of Science database using the terms “forest AND resilience”. From that list we selected articles according to the following criteria: 1) reported a vegetation response metric of plant performance after a disturbance event in the field; and, 2) compared post-disturbance plant performance under two contexts. We considered context of recovery to refer to the recovery process under two different sets of conditions: different resource levels along a natural gradient (moisture, fertility, diversity), different management practices (fertilization, biomass reduction), or differences in the number of disturbances or in the severity of the disturbance (Table 1).

**Table 1:** Partial list of the variables extracted and the categories and metrics considered. For a full list and descriptions see Supporting information (S1 Text).

Variable	Description	Categories/metrics
**Disturbance**	Type	clearing, drought, erosion, fire, flood, herbivory, logging, wind
**Forest type**	Forest origin	natural, plantation
**Type of system**	Management status during recovery	**M**: management, management practice implemented
		**N**: natural, no intervention
**Context of recovery**	Conditions under which recovery took place. Comparisons were made between low levels (control) and high levels (treatment) of resources/ management/disturbance severity/second disturbance	Post-disturbance **biomass reduction** (M): thinning, clearing, salvage logging, burning, herbicide treatments, tilling
		**Diversity** gradient (M,N): species richness
		**Fertility** gradient/treatment (M,N): additions, site index
		**Moisture gradient** (N): precipitation, TMI, PDSI
		Exposure to a **second disturbance** (M,N): fire, thinning, clearing, herbivory, invasive species, soil compaction
		Level of disturbance **severity** (M,N): intensity, duration, frequency
**Response metric**	Post-disturbance plant performance (responses are compared between two contexts of recovery).	**Abundance**: density, ground/canopy cover, dominance, basal area, NDVI, LAI
		**Change**: LAI, species composition, productivity, richness, cover
		**Diversity**: species richness, floristic index, community dissimilarity (Sorensen), rarefied species richness, diversity index (Shannon, Simpson’s, Weiner)
		**Growth**: BA, height, biomass, NPP, DBH, LAI, NDVI
		**Reproduction**: no. cones, no. seeds
		Resilience*: post-drought growth/pre-drought growth
**Vegetation strata**	Vegetation categories sampled	Adult trees, all, bryophytes, epiphytes, forbs, graminoids, pteridophytes, saplings, seeds, seedlings, shrubs, understory, vines, and woody

N: natural system; M: managed system; TMI: topographic moisture index; PSDI: Palmer drought severity index; NDVI: normalized difference vegetation index; LAI: leaf area index; BA: basal area; DBH: diameter at breast height; NPP: net primary productivity.

*We included an index of resilience as one of the recovery responses because this is a commonly used metric in the literature. Several resilience indices have been described, in general they compare disturbance or post-disturbance performance with pre-disturbance tree radial growth, e.g., resilience: drought growth/pre-drought growth

For each study we extracted information on geoclimatic variables (e.g., location, elevation, temperature and precipitation). We then recorded site and disturbance information, qualitative information on the conditions of recovery and on the type of response metric (Table 1). We also gathered quantitative data on these variables, i.e., sample size, mean and SD or SE (data from figures were extracted using: https://automeris.io/WebPlotDigitizer/). For a full list of variables extracted and the categories and metrics considered see Supporting information (S1 Text).

### Data classification and effect size calculation

**Fig 1:**
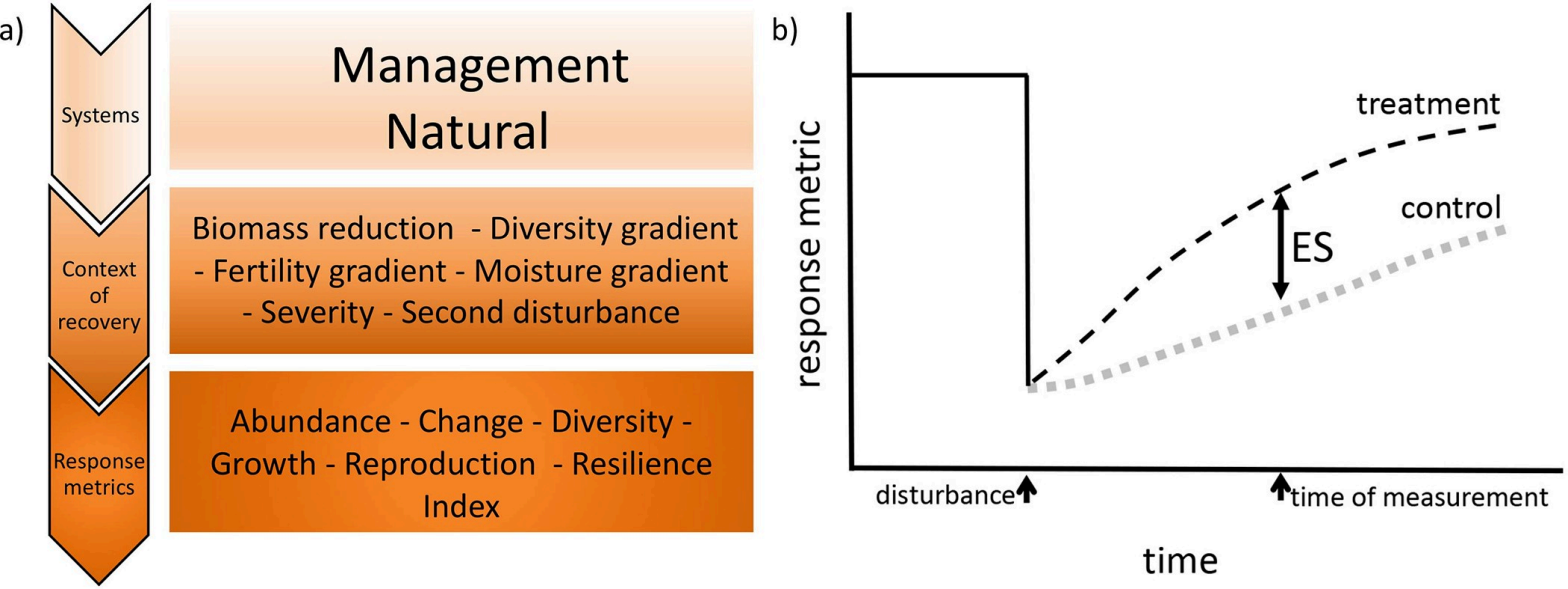
Representation of the analysis. a) Representation of the hierarchical analysis of effect size. b) Visual representation of the effect size (ES) calculation. Most studies do not describe initial conditions, thus, the pre-disturbance line is hypothetical; these are likely to be different between control and treatment when referring to natural gradients (diversity, fertility and soil moisture).

Data were classified using a hierarchical structure (Fig 1A). First, data points were divided into two systems: under management or natural, these two groups included both wild forests and plantations that were either managed (management) or left to recover with no intervention (natural). Management in most studies was aimed at improving tree growing conditions, i.e., reducing competition, adding fertilized, reducing herbivory, with a few studies where management was aimed at reducing fuel load, i.e., clearing of understory vegetation. Within each system, data were assigned a context under which recovery took place, leading to six distinct categories (Table 1). Next, plant performance, reported under two different conditions, were classified into six metrics of recovery (Table 1).

The advantage of carrying out a meta-analysis is that we were able to simultaneously consider many different types of response metrics by estimating effect size. For that, we compared responses in the low range of the natural resource or managerial gradients (low moisture site, low fertility site, low diversity site, low severity disturbance, no or low degree of post-disturbance management, no second disturbance; for the purpose of our analyses we considered this to be the control response; Fig 1B) with those in the high range of the gradient (high moisture site, high fertility site, high diversity site, high severity disturbance, high degree of post-disturbance management, incidence of a second disturbance; treatment response). Given the diversity of response variables, with positive, negative and mixed values, we estimated effect size, ES, as: $ES=\frac{\left(treatment\;response-control\;response\right)}{absolute(average\;tretament\&control)}$. This estimate prevents any manipulation of the data (e.g, log transformations, additions) that could bias results, and it is still highly correlated with more traditional metrics (e.g., natural log of the ratio, differences). Calculations follow [47,48] and account for sample size (i.e., reported variances were weighed by sample size [49]). See Supporting information for detailed description of ES calculations (S2 Text).

### Data analysis

We analyzed the calculated values of ES using a hierarchical approach. ES was estimated at two levels: 1) for each system*context of recovery*response metric combination, which were nested within 2) each system*context of recovery combination. We also included study random effects (see Supporting information for detailed description; S2 Text). Pre-disturbance conditions were not accounted for since these were rarely reported. For forests undergoing a second disturbance, time between disturbances was not included because most studies did not provide that information.

In addition, we carried out extensive exploratory data analysis to determine what might have affected the variability we observed in ES (e.g., type of disturbance, biome, climate). Only a few trends emerged, these were associated with recovery along moisture gradients, biomass reduction treatments, and severity of the disturbance.

We analyzed the response to moisture gradients (only for natural systems) as a function of the moisture levels of the study’s region. We aimed at understanding if the effects of moisture gradients on resilience depended on the climatic conditions of the region. For example, moisture gradients may play a more important role on resilience in dry regions than in wet areas. We calculated a variant of the De Martonne humidity-aridity index (DMI) that includes temperature and precipitation, DMI: annual precipitation (cm)/July average temperature (°C) (January for southern hemisphere) [50]. We also included study random effects and years since disturbance as the magnitude in ES may vary over time. Since data exploration indicated that responses might vary by biome, analyses were carried out for each biome.

To test whether the type of disturbance affected the outcome of post-disturbance biomass reduction practices we analyzed the data available (only managed systems) as a function of disturbance type and study random effects. We did not have enough observations to test all combinations of disturbance type and response metrics.

Our final analysis focused on assessing if disturbance severity affected vegetation responses. We analyzed ES as a function of strength of severity. To be able to assess the effect of severity across the variety of studies surveyed, we estimated strength of severity using the same approach as ES ($Severity=\frac{\left(treatment\;severity-control\;severity\right)}{absolute(average\;treatment\&control)}$) [47]. Since severity seemed to affect vegetation strata differently (known from our exploratory data analysis), we estimated the effect of *Severity* on ES for several vegetation stratum (only ‘all strata’, ‘adult trees’ and ‘seedlings’ categories had enough data, others had much fewer data points, < 6). Study random effects were also added. Detailed description of all the analyses is provided in the Supporting information (S2 Text).

To estimate parameters from these hierarchical mixed models we carried out Bayesian analyses; this approach easily accommodates the multilevel structure of the models, the mixed effects, and any issues with unbalanced or missing data [51]. Analyses were run in OpenBUGS [52], with three chains, for 20,00 iterations. Only the last 10,000 iterations, after convergence, were used and thinned to estimate parameter posterior means, variances, and covariances (see Supporting information for analyses code (S2 Text)).

## Results

**Fig 2:**
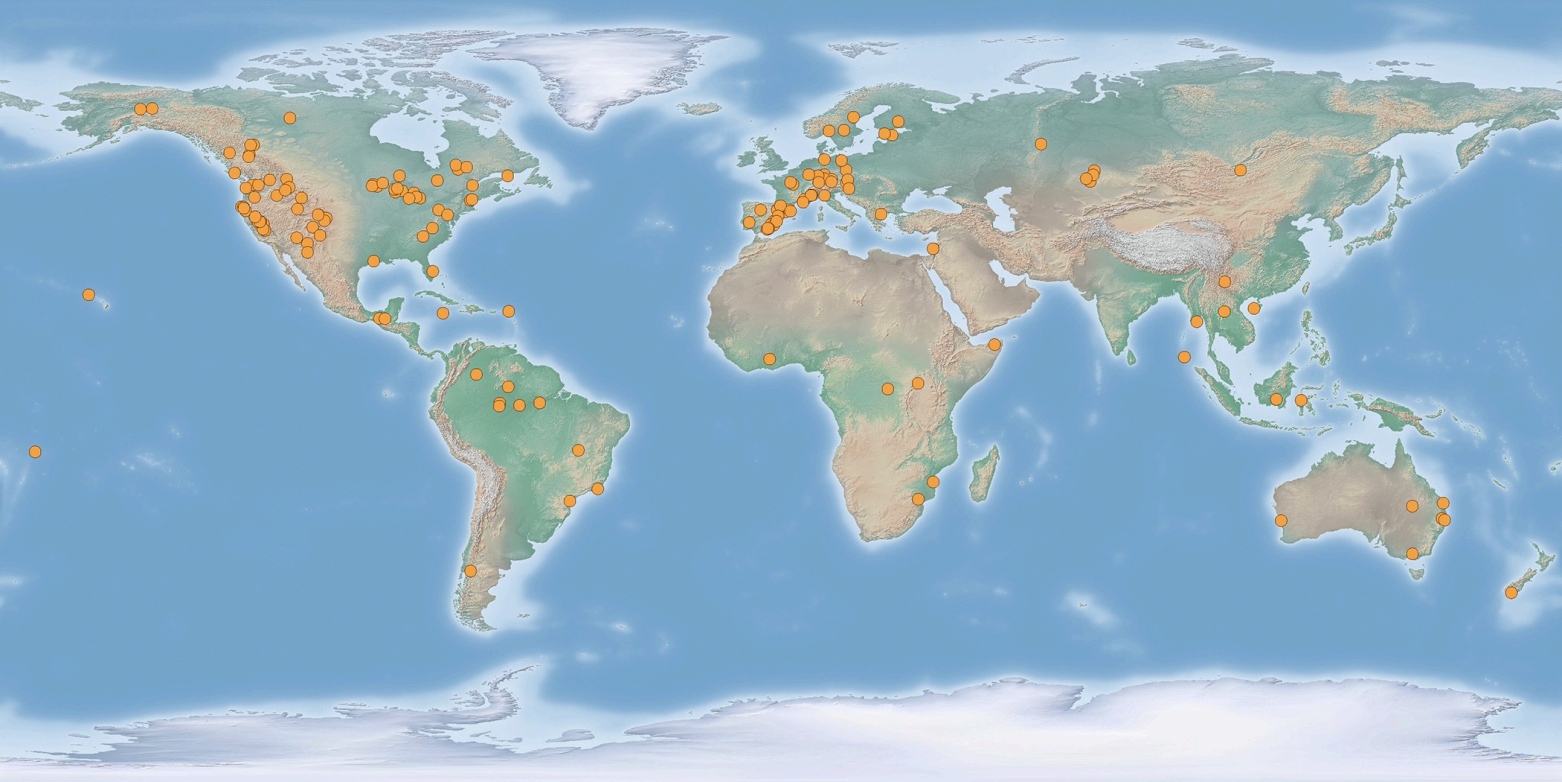
Geographic distribution of the data. Locations of the 724 data points used in the analyses.

Our partial search of the scientific literature on forest resilience generated ~2,500 references (up to the search date). After applying our selection criteria, 156 articles were included in the final analysis (Supporting information, S3 Text). Data extraction resulted in 724 data points, some articles reported several individual species responses, others reported several metrics of recovery, and a third group assessed recovery at different sites (see Supporting information for data, S1 File, and data distribution graphs,S1 Fig). Most reported data were distributed across Western Europe and the USA (Fig 2). The number of observations available for each combination of system and context of recovery varied widely (from 4 to 203). The data set contained 112 observations of recovery along moisture gradients (8 observations for boreal forests, 21 in Mediterranean forests, 69 in the temperate region, and 14 in the wet tropics); 193 observations were relevant to the analysis of the effects of post-disturbance biomass reduction treatments; and, there were 166 observations testing the effects of disturbance severity on resilience (all strata 42 observations, adult trees 72 observations, and seedlings 51 observations). Only 21 entries explicitly reported recovery of exotic species, thus we did not analyzed separately. Parameter values, means, SDs and 95% credible intervals from the analyses can be found in the Supporting information (S1 Table).

### Hierarchical analysis of ES

**Fig 3:**
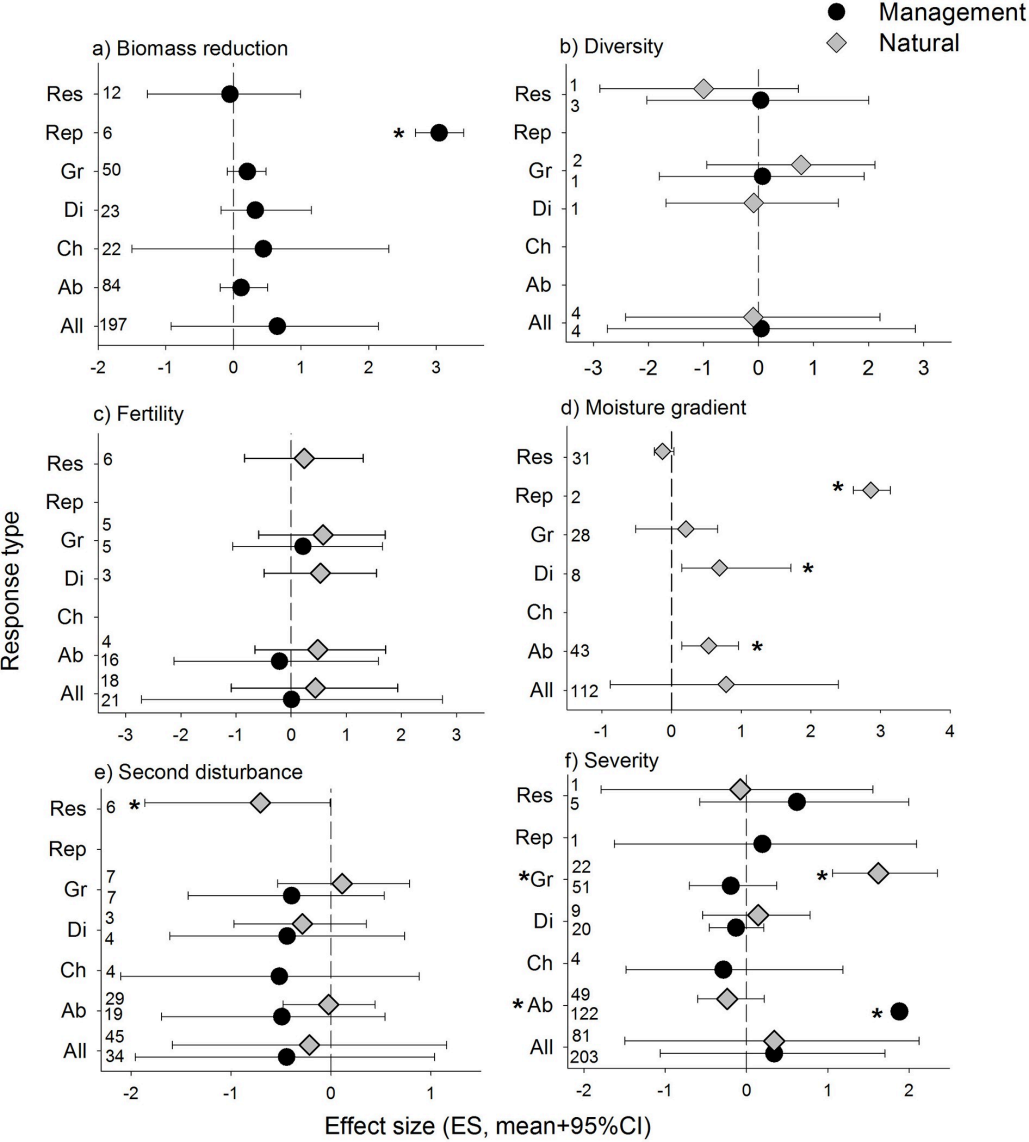
Results from the hierarchical analysis. Estimated effect size for each combination of system*context of recovery (All) and for each response metric (Ab: abundance, Ch: change, Di: diversity, Gr: growth, Rep: reproduction, Res: resilience). Numbers indicate number of observations available. * Indicates statistical significance, i.e., credible intervals (CI) do not intercept the zero line, or management and natural do not intercept with each other.

The estimated ES for each combination of system (management and natural) and context of recovery (biomass reduction, diversity, fertility, moisture, second disturbance and severity) were very broad (‘All’ in Fig 3). It is only when we examined specific response metrics (abundance, change, diversity, growth, reproduction and resilience) that we find significant effects on recovery. Biomass reduction treatments have an overall positive effect on plant performance, but this effect was only significant for reproduction (Fig 3A). Diversity and fertility gradients did not seem to make a significant difference in how forests recover after disturbance (Fig 3B and 3C); also note the very low number of diversity studies. Higher moisture levels had positive effects on abundance, diversity and reproduction (Fig 3D). The response to a second disturbance was statistically significant, and negative, only for the resilience index (Fig 3E). Effect sizes for disturbance severity were rather variable, with significantly positive responses for abundance in managed systems and for growth in natural systems (Fig 3F).

### Analysis within moisture gradients, biomass reduction treatments and severity gradients

**Fig 4:**
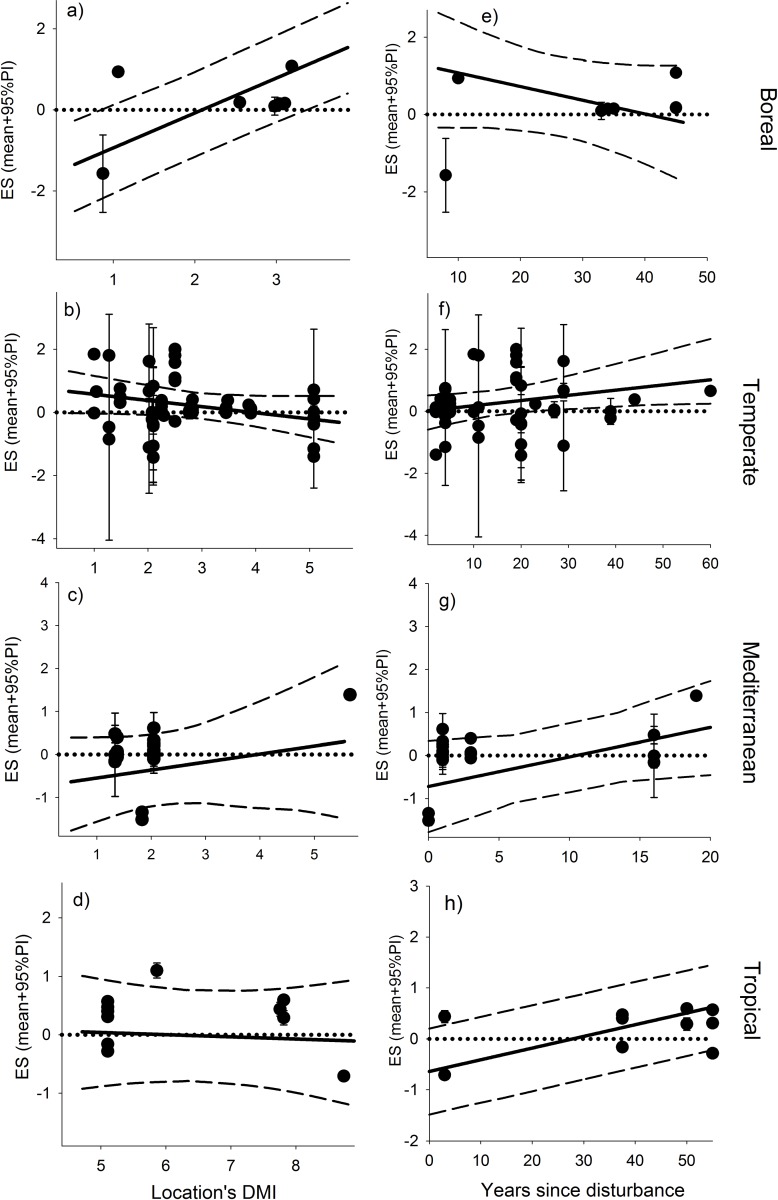
Results from moisture gradient analysis. Changes in effect size as a function of De Martonne humidity-aridity index (DMI) (a-d), and as a function of years since disturbance (e-h), for each of the sampled biomes. Dots represent estimated ES for each observation (mean and SD). Lines indicate predicted ES (mean and 95% predicted interval [PI]). Predictions were estimated at the average number of years since disturbance in that biome (a-d) or at the average DMI value in that biome (e-h). Predicted intervals that do not intercept with zero are considered statistically significant; an asterisk indicates the slope parameter is statistically significant (95% CI does not overlap with zero).

The effect of moisture gradients on forest recovery varied among biomes (model goodness of fit, predicted *vs* observed R^2^ was 0.62). In boreal systems, the effect of higher water availability increased with the humidity-aridity index (DMI) of the site (slope parameter; Fig 4A). Also in this biome, effect sizes sifted from negative to positive (95% predicted intervals [PI] do not overlap with zero) from dry to wet regions, while the temperature biome had positive, and significant, ES values in the drier regions (Fig 4B). Effect sizes significantly increased with years from disturbance in tropical forests (slope parameter, Fig 4H). But ESs were only statistically significant with more years since disturbance in the temperate biome (95% PI; Fig 4F).

**Fig 5:**
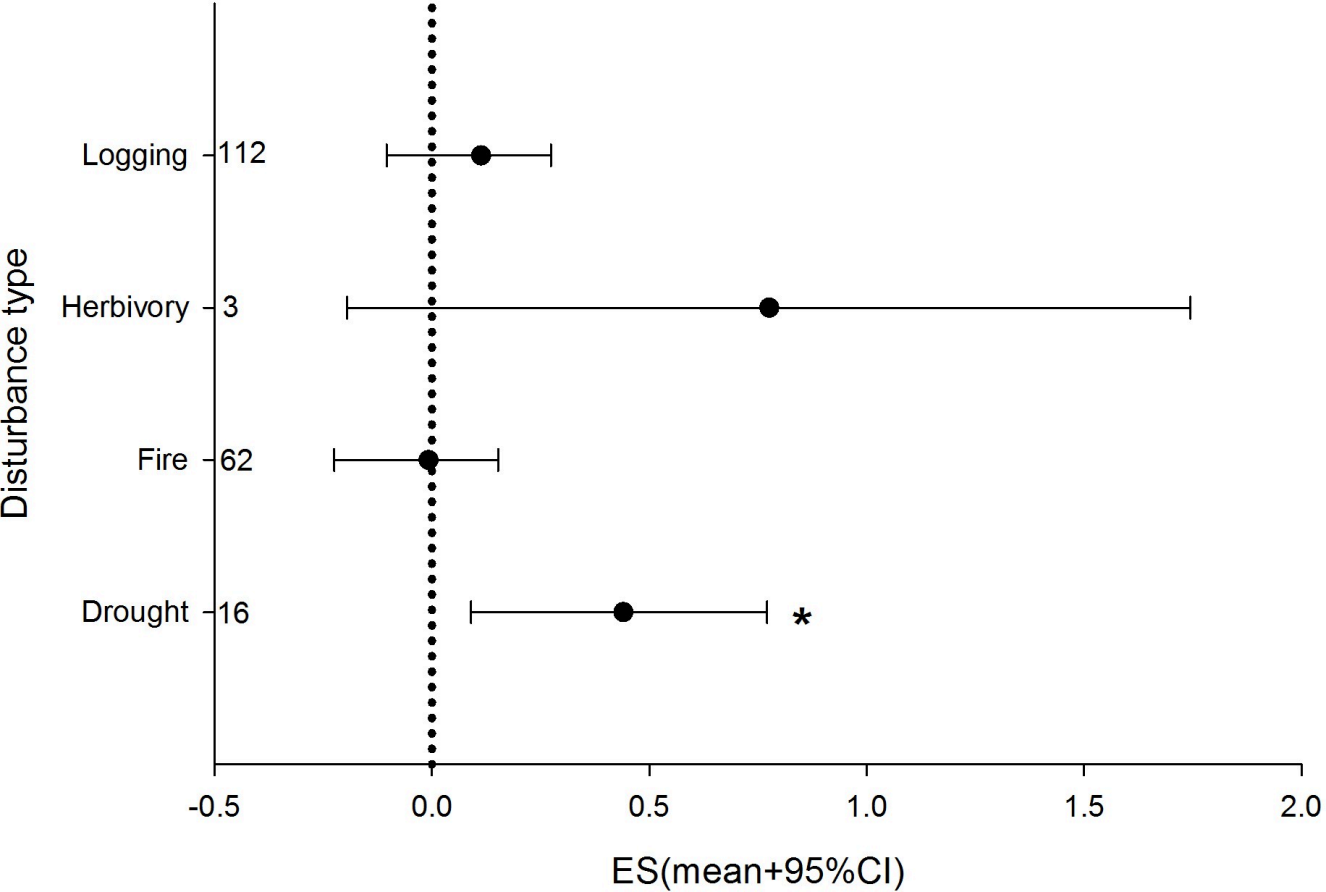
Results from the analysis of biomass reduction treatments across disturbance types. Credible intervals that do not intercept with zero are considered statistically significant (*).

**Fig 6:**
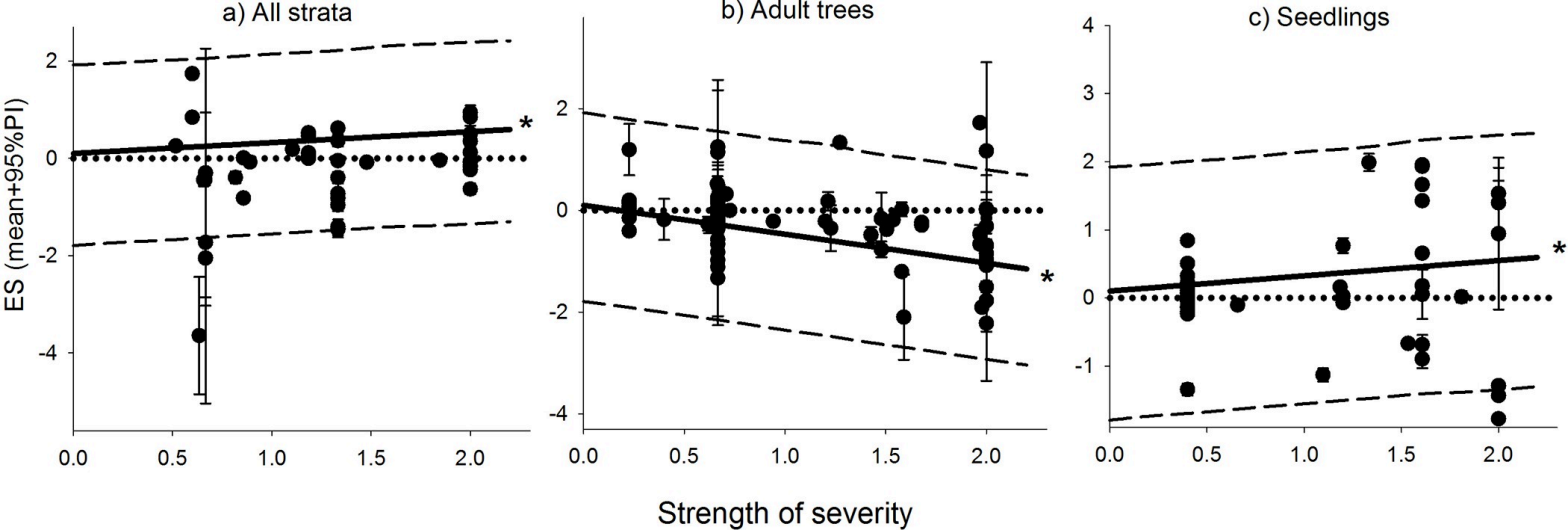
Changes in effect size as a function of severity strength for three vegetation strata. Dots represent estimated ES for each observation (mean and SD). Lines indicate predicted ES (mean and 95% predicted interval [PI]). Predicted intervals that do not intercept the zero line are considered statistically significant, an asterisk indicates the slope parameter was statistically significant (95% CI does not overlap with zero).

The analysis of the post-disturbance biomass reduction treatments show how these practices were only associated with positive vegetation responses if the disturbance event was a drought (Fig 5). Higher disturbance severity had a significant and positive effect on ES for seedlings and all strata categories, but a negative effect, also significantly, for adult trees (slope parameters; Fig 6; model goodness of fit, predicted *vs* observed, R^2^ was 0.42).

## Discussion

Since both disturbance regimes and recovery mechanisms will be affected by global change [3], understanding forest ecosystems’ capacity to recover will be essential for the developing of sound conservation and management plans. The cross section of the literature on forest resilience we reviewed provided insight into broad patterns of forest recovery in response to natural conditions or management practices, it also highlighted conditions that could jeopardize future resilience—information that can now be considered in conservation and management planning and inform future research and analyses. But most importantly, this review identified critical knowledge gaps that will need to be addressed if we aim at promoting future forest resilience. Our review showed that post-disturbance biomass reduction treatments, a widespread practice to favor growth in managed forests, only increased recovery after a drought. As expected, high soil moisture had a beneficial effect on recovery. We found that the effect of higher moisture on recovery increased with regional humidity in boreal forests, while it was only significant, and positive, in dry areas in the temperate biome. Our analyses also show that the beneficial effects associated with higher disturbance severity were mainly at the seedling stage. Although this information can now be used to assess resilience across areas and plant stages, the most relevant aspect of our review were the knowledge gaps we identified. For example, there is little information about the role of biodiversity in the recovery process, or, about the level of resilience that would be necessary under future environmental changes (e.g., increase drought conditions, fire frequency).

### Impact of resource gradients

The conditions under which plant growth takes place after disturbance play a critical role on the speed of recovery [53]. Thus, we expected that higher resource conditions would be associated with higher response rates. However, the effects of the resource gradients tested, moisture and fertility, were not always relevant (Fig 3). Only abundance, diversity, and reproduction had a significantly positive response to higher water availability. This outcome likely reflects better conditions for recruitment under more humid soils [54,55,56], and more resources for reproduction [57].

As expected, the responses to soil moisture gradients we examined showed that most recovery metrics were higher at the wetter extreme of the moisture gradient (Fig 3), and point at a potential decrease in forest resilience under the predicted warmer and drier conditions forecasted under climate change [58,59]. The other resource gradient we examined, fertility, did not show any trends (Fig 3). And, although we did not test the combined effects of drought and fertilization, research suggests that the negative effects of drought could be mitigated by higher nutrient levels [60], making fertilization, if viable, a management practice to improve forest resilience to future droughts.

The analysis of moisture gradient data also showed that water availability may play a different role across regions with different humidity-aridity indices (Fig 4). These differences were quite remarkable in boreal and temperate forests, although the trends were opposite from each other. As we have expected, increased moisture had a positive effect in recovery, but only in the drier regions in temperate biomes (Fig 4B, PI intervals do not cross zero). However, in boreal forest, only regions with high humidity-aridity indices experience a positive effect of moisture availability on recovery, while the effect of moisture was negative in drier regions (Fig 4A). This information is useful because it could help identify which forest stands may be at a higher risk of slow recovery after a drought. Logging operations usually target wetter sites to be harvested more frequently, but this approach could be more effective in regions with a drier climate within temperate forests and in regions with a more humid climate within boreal forests (a note of caution is warranted here since the number of studies considered was low).

### Impact of forest management practices

Forest management practices involving post-disturbance biomass reduction, fertility treatments, or the implementation of a second disturbance (e.g., burning, salvage logging) are common [61,62]. However, as our analysis revealed, they are not always effective at increasing vegetation recovery after disturbance (Fig 3). Thus, if resilience is the goal, these practices should be tested and adjusted to improve the outcome of the intervention. Still, biomass reduction was particularly effective at ameliorating the effects of drought (Fig 5). This effect has been extensively documented [32] as thinning operations are widely performed in forests [31,63,64]. The same mechanism that improves growth under biomass reduction treatments—i.e., reduced competition for light—also reduces competition for water under drought conditions [65,66]. Thus, given the increased likelihood of droughts under global warming [38], this practice may have to be implemented in areas that until now have not experienced water scarcity.

Furthermore, identifying the most drought susceptible areas in the landscape will be critical to optimize management. Across the landscape, drier sites are populated by either drought-adapted species or drought-acclimated populations, while wetter sites usually host mesic, drought-intolerant, species [67,68]. These latter sites have developed deeper and richer soils that have high water holding capacity, factors that can alleviate the impact of drought. However, the interaction between tree drought tolerance and a site’s ameliorating conditions for determining the outcome of a drought event is still unknown. Our results shed some light on this issue. Among the response metrics to soil moisture gradients, only the resilience index considered pre-disturbance plant performance, and accounted for individual acclimation to growing conditions. The marginally negative ES estimated from the resilience index data (Fig 3D), indicates that, within a species, trees growing at higher moisture conditions are more affected than those acclimated to drier environments [12]. Therefore, the ability to account for site-specific drought impacts will not only identify susceptible areas that can be targeted for management, but it will also improve estimates of regional forest productivity under climate scenarios.

### Impacts of multiple disturbances and severity

Since the incidence of some disturbances is likely to increase under future environmental conditions [38], understanding the effects of multiple disturbances will be fundamental in the assessment forest resilience. Compounded disturbances may, not only compromise resilience, but also cause a change in ecosystem stage [18]. A decline in forest resilience to increasing wildfires has been documented in areas of western North American [69]. And, tropical forests are experiencing lower levels of resilience due to continued deforestation [70]. Furthermore, current climatic trends may alter post-disturbance conditions and further affect recovery after recurrent disturbances [2]. Although ES tended to be negative (Fig 3), our results were inconclusive with respect to the effects of a second disturbance. However, we did not account for time between disturbances, information was not always provided, thus, we could not assess the potential effects of changing disturbance frequency which are likely to be negative [18].

Following the same dynamics of higher resource availability after disturbance, increased disturbance severity was associated with higher abundance of recruitment stages (Fig 6). This again is likely the result of reduced competition for light and soil resources. However, even if recruitment stages benefit, later developmental stages, i.e., trees left behind, did not. From our analysis we cannot assess the overall effect of disturbance severity on whole ecosystem, but we can assert a note of cation about evaluating the impact of disturbance severity on the basis of only one developmental stage.

## Conclusions

Forest ecosystems provide a large array of provisioning, regulating, sustaining and cultural services to humans [71]. In future decades, forest’s preservation will depend on the capacity of these systems to recover after disturbance. The insights from our review, although informative to assess general areas of low and high resilience, are limited in their scope. We only sample a fraction of the available literature on forest recovery, limiting our analysis to broad patterns of how intrinsic and extrinsic factors may affect forest recovery. But, most relevant, we identified several critical knowledge gaps.

Despite the existence of an extensive body of literature on the effects of species diversity on forest productivity [72,73,74,75,76] and resistance to pest outbreaks [77,78], we found very few studies considering sites with different diversity levels. Interestingly, there is almost no empirical or experimental evidence about how diversity may affect plant performance after disturbance (but see [23,25,79]). This highlights an area for future research, as diverse ecosystems are thought to be more resistant to disturbances caused by insect pests, diseases, and drought [21,78,80,81]. If recovery were also to be affected by diversity, then management practices geared toward increasing diversity should be promoted [82].

Only a partial geographic area was well represented in the data (North America and Western Europe; Fig 2), which limited the representation of large forest biomes, i.e., tropical and boreal. Finally, remarkably absent were any insights that could have addressed the following questions: 1) should resilience be based on the recovery of a particular species or developmental stage, or on the functionality of the whole ecosystem? 2) What level of resilience is acceptable? Complete recovery to previous conditions, less, or recovery to a different stage [83]? 3) Is the level of past resilience enough or should we aim at higher levels, i.e., transformative resilience [84]? And, 4) because forests will be recovering under a changing environment, and they are slow to adapt, could we accelerate that process with management practices? Particularly, we will need to address fundamental issues like deciding how to frame resilience, determining what level of resilience is necessary to cope with future environmental changes, and considering the possibility of transformative resilience. Since changes in environmental conditions may be too fast for forests to adapt to, these are all issues that should be taken into consideration in the conservation and management of forest ecosystems.

## Supporting information

S1 Text Full list of variables extracted. List of variables extracted and metrics considered.
(PDF)

S2 Text Analytical methods. Description of effect size calculations and analyses. Analyses computer code.
(PDF)

S3 Text List of included studies. Publications included in the final analyses.
(PDF)

S1 File Data used in the analyses. Data extracted from selected publications and used in the analyses.
(XLSX)

S1 Fig Distribution of observations. a) Proportion of the data under each disturbance for the two systems. b) Proportion of the data under each disturbance and context combination for the two systems. Numbers in parenthesis indicated number of observations.
(PDF)

S2 Fig PRISMA flow diagram. (PDF)

S1 Table Parameter values, means, SD and 95%CI. Numbers in bold indicate statistically significant effect sizes or covariates, different letters indicate statistically significant differences between biomes or strata (i.e., 95% credible intervals [CI] do not intersect with zero).
(PDF)
