# Supplementary material for: Forest resilience under global environmental change: Do we have the information we need? A systematic review
Source: PLoS One. 2019 Sep 12;14(9):e0222207. doi: 10.1371/journal.pone.0222207 (PMC6742408; doi:10.1371/journal.pone.0222207)
Supplement: S1 Text — List of variables extracted and metrics considered. (PDF) [file pone.0222207.s001.pdf]

Supplemental information S1 Text

List of variables extracted and the categories and metrics considered

Longitude (decimal, if several sites, central location)
Latitude (decimal, if several sites, central location)
Continent
Country
City (nearest, if several sites were included, we used the central location)
Elevation (m, if several sites were included, we used the central location)
Biome (boreal, mangrove, Mediterranean, subtropical, temperate, tropical)
Precipitation (mm; if not reported we carried out a search for conditions in the closest city. If several sites were included, we used the central location)
January average temperature (°C; if not reported we carried out a search for conditions in the closest city. If several sites were included, we used the central location)
July average temperature (°C; if not reported we carried out a search for conditions in the closest city. If several sites were included, we used the central location)
Type of forest (natural, planted, mixed)
Year of disturbance
Year of measurement
Years since disturbance
Disturbance (clearing, drought, erosion, fire, flood, herbivory, logging, wind)
Type of system (management, natural)
Context of recovery (biomass reduction, diversity gradient, fertility, intrinsic [genetic differences], moisture gradient, second disturbance, severity).
Control context of recovery description (low intensity, low resources, no-management, no second disturbance).
Treatment context of recovery description (high intensity, high resources, management, second disturbance).
Unit of context of recovery (e.g., tree density, drought severity index, number of fires).
Response metric description (abundance/cover, change from previous condition, diversity, growth, reproduction, resilience*)
Units of response variable
Vegetation strata (adult trees, all, bryophyte, epiphyte, forbs, graminoids, pteridophytes, saplings, seeds, seedlings, shrubs, understory, vines and woody).
Source of response data (table, figure, text, appendices).
Control sample size.
Control context of recovery mean.
Control context of recovery SD.
Control response mean.
Control response SD.
Treatment sample size.
Treatment context of recovery mean.
Treatment context of recovery SD.
Treatment response mean.
Treatment response SD.

[a]We included and index of resilience as one of the recovery responses because this is a commonly used metric in the literature (e.g., Lloret et al. 2011, D'Amato et al. 2013). Several resilience indices have been described, in general they compare disturbance or post-disturbance performance with pre-disturbance tree radial growth, e.g., resilience: drought growth/pre-drought growth.

D'Amato, A. W., J. B. Bradford, S. Fraver, and B. J. Palik. 2013. Effects of thinning on drought vulnerability and climate response in north temperate forest ecosystems. Ecological Applications **23**:1735-1742.

Lloret, F., E. G. Keeling, and A. Sala. 2011. Components of tree resilience: effects of successive low-growth episodes in old ponderosa pine forests. Oikos **120**:1909-1920.
