# Supplementary material for: Forest resilience under global environmental change: Do we have the information we need? A systematic review
Source: PLoS One. 2019 Sep 12;14(9):e0222207. doi: 10.1371/journal.pone.0222207 (PMC6742408; doi:10.1371/journal.pone.0222207)
Supplement: S2 Text — Description of effect size calculations and analyses. Analyses computer code. (PDF) [file pone.0222207.s002.pdf]

Supplemental information S2 Text

Description of effect size calculations, detailed data analysis, and computer code used for the analyses.

Effect size calculations

Because we collected data from very different types of variables, conventional calculation of effect size (ES), e.g., natural log of the ratio treatment/control, would have required data transformations that could have impacted the results. For example, some of the data points were zero and some included negative values for only one of the responses which would have produced an ES with erroneous signs (positive when the actual effect was negative). Instead, we used a calculation of the effect size that does not required data transformation to deal with those issues and that always produce an ES with the right sign. Furthermore, these values are still highly correlated with the natural log ratio (Sorte et al., 2012; Ibáñez et al., 2014). Effect size was estimated as:

$$ES = \frac{(treatment - control)}{absolute\ value\ (average(treatment, control))}$$

This is also a relatively conservative estimate of ES, values varied between -2 and 2, while calculations of the natural log ratio varied between -5.2 and 6.4. The correlation between our ES estimates and those using the natural log ratio was 0.99 (based on 591 observations with ES values between -1.5 and 1.5). To estimate the mean and variance around the effect size we run simulations (equivalent to bootstrapping) using the reported mean and SD or SE values of the response metrics, we also included sample size in the calculations to give more weight to observations with larger sample sizes (Gurevitch & Hedges 2001).

Fig.SM3. Graphical representation of ES (x axis) and their associated variability (SD, y – axis). Variability estimates account for reported sample size.

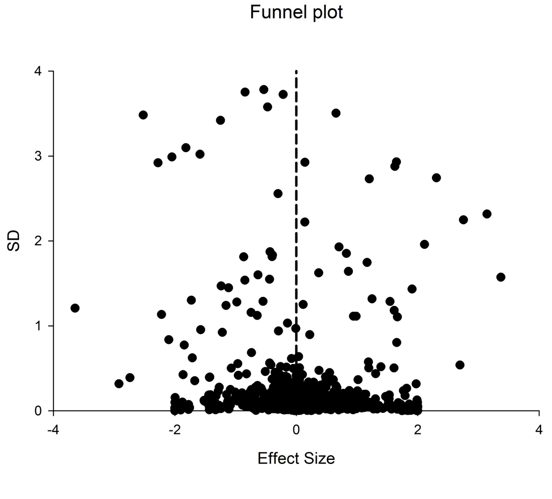

Gurevitch, J. & Hedges, L.V. (2001) Meta-analysis - combining the results of independent experiments. *Design and Analysis of Ecological Experiments* (eds S.M. Scheiner & J. Gurevitch). Oxford University Press, Oxford.

Ibáñez, I., Katz, D.S.W., Peltier, D., Wolf, S.M. & Connor Barrie, B.T. (2014) Assessing the integrated effects of landscape fragmentation on plants and plant communities: the challenge of multiprocess–multiresponse dynamics. **102,** 895.
Sorte, C.J.B., Ibáñez, I., Blumenthal, D.M., Molinari, N.A., Miller, L.P., Grosholz, E., … Dukes, J.S. (2012) Poised to prosper? A cross-system comparison of climate change effects on native and non-native species performance. *Ecology Letters,* **16,** 261-270.

Data analysis

In order to compare natural and managed systems and to differentiate between conditions that were expected to improve resilience (increases in moisture, fertility or diversity, and biomass reduction treatments) *versus* those that were expected to reduce it (increase in disturbance severity and occurrence of a second disturbance), we analyzed the calculated values of ES, mean (ES) and SD (σ) using the following hierarchical approach. For each observation i, ES_i was estimated for each system (natural or managed), context of recovery (moisture, fertility, diversity, biomass reduction, severity, second disturbance) and response metric (abundance, change, diversity, growth, reproduction, resilience). We also included study random effects, SRE.

Likelihood: $ES_i \sim Normal(ESm_{i,} \sigma_i^2)$

Process model: $ESm_i = ES1_{system(i),context(i),response(i)} + SRE_i$

Parameter ES1 was then estimated for each combination of system and context of recovery, $ES1_{system,context,response} \sim Normal\left(ES2_{system,condition}, \sigma_{system,condition}\right)$.

We carried out additional analysis to determine what might have affected the variability we observed in ES. From the six contexts of recovery, we had enough data (more than 100 observations) to analyze three of them: moisture gradients, biomass reduction treatments and disturbance severity.

We analyzed the response to moisture gradients (data were only available for natural systems) as a function of the moisture levels of the study's region. The purpose of this approach was to understand if the effects of moisture gradients on resilience depended on the climatic conditions (moisture regime) of the region. For example, moisture gradients may play a more important role on resilience in dry regions than in wet areas. For that we calculated a variant of the De Martonne humidity-aridity index (DMI) that includes temperature and precipitation, DMI: annual precipitation (cm)/July average temperature (°C) (January for southern hemisphere locations) (De Martonne 1926). We also included study random effects (SRE) and years since disturbance (YSD) as the magnitude in ES may vary over time. Since data exploration indicated that responses might vary by biome, parameters were estimated for each biome represented in the data (boreal, temperate, Mediterranean and tropical). DMI and YSD were standardized for the analysis.

Likelihood: $ES_i \sim Normal(ESm_{i,} \sigma_i^2)$

Process model: $ESm_i = \beta1_{biome(i)} + \beta2_{biome(i)} DMI_i + \beta3_{biome(i)} YSD_i + SRE\beta_i$

To test whether the type of disturbance affected the outcome of post-disturbance biomass reduction practices we analyzed the data available (only from managed systems) as a function of disturbance type and study random effects (SRE).

Likelihood: $ES_i \sim Normal(ESm_{i,} \sigma_i^2)$

Process model: $ESm_i = \alpha_{disturbance(i)} + SRE\alpha_i$

Finally, we analyzed whether severity of the disturbance affected vegetation responses. We analyzed the available data (for natural and managed systems) and estimated ES as a function of the treatment size (TS; a measurement of severity strength). TS was estimated using the same approach as ES ($TS = \frac{(treatment-control)}{abs(average)}$). Since severity seemed to affect vegetation strata differently (known from our exploratory data analysis), we estimated the effect of TS on ES as a function of vegetation stratum (only 'all strata', 'adult trees' and 'seedlings' categories had enough data, others had much fewer data points, < 6). Study random effects were also added and centered to aid with the convergence of parameters.

Likelihood: $ES_i \sim Normal(ESm_{i,} \sigma_i^2)$

Process model: $ESm_i = \gamma_{vegetation(i)} TS_i + (SRE\gamma_i - meanSRE\gamma)$

Parameters for all analyses were estimated from non-informative prior distributions:

$$ES2_{system,context}, \alpha_{disturbance}, \beta_{biome,} \gamma_{vegetation} \sim Normal(0{,}10000)$$

$SRE *_i \sim Normal(0, \sigma_{SRE*}^2)$ and $\sigma_{SRE*}^2 \sim Uniform(0{,}100)$

Analyses were run in OpenBUGS (Thomas et al. 2006), with three chains, for 20000 iterations. Only the last 10000 iterations, after convergence, were used and thinned to estimate parameter posterior means, variances, and covariances.

De Martonne, E. (1926) Areisme et indice d'aridite. *Academie des Sciences, Comptes,* **182,** 1395–1398.

Thomas, A., O'Hara, R., Ligges, U. & Sturts, S. (2006) Making BUGS Open. *R News,* **6,** 12-17.

Code in OpenBUGS-Effect size calculations:

```
model{

for(i in 1:726){

ct[i]<-Nc[i]/pow(sdc[i],2) #precision control response, correcting for sample size
tt[i]<-Nt[i]/pow(sdt[i],2) #precision treatment response, correcting for sample size

C[i]~dnorm(meanc[i],ct[i])
T[i]~dnorm(meant[i],tt[i])

m[i]<-abs((T[i]+C[i])/2)
ES[i]<-(T[i]-C[i])/m[i]))
}

}#end model
```

Code in OpenBUGS-Effect size hierarchical analysis:

```
model{

for(i in 1:726){
ESmean[i]~dnorm(ESm[i],EStau[i])
ESm[i]<-ES1[Driver[i],treat[i],res[i]]+SRE[study[i],res[i]]
}
```

```
#priors
  for(i in 1:2){ #system
     for(j in 1:7){ #condition
          ES2[i,j]~dnorm(0,0.1)
          T2[i,j]<-1/var2[i,j]
           var2[i,j]~dunif(0,10)

        for(k in 1:6){#respose
         ES1[i,j,k]~dnorm(ES2[i,j],T2[i,j])
}}}

for(r in 1:6){
for(i in 1:157){ SRE[i,r]~dnorm(0,T) }}
T<-1/V
V~dunif(0,100)

} #end model
```

Code in OpenBUGS-Effects of Moisture gradients:

```
model{
#missing values
DMI[36]~dunif(0.86,8.74)

for(i in 1:112){

DMIS[i]<-(DMI[i]-mean(DMI[]))/sd(DMI[])
yearsS[i]<-(years[i]-mean(years[]))/sd(years[])

ESmean[i]~dnorm(ESm[i],EStau[i])
ESm[i]<-a[Biome[i]]+b[Biome[i]]*DMIS[i]+c[Biome[i]]*yearsS[i]+SRE[study[i]]

}

#for(r in 1:6){
for(i in 1:151){ SRE[i]~dnorm(0,T) }  #}
T<-1/V
V~dunif(0,10000)

for(d in 1:6){
a[d]~dnorm(0,0.0001)}
for(i in 1:6){b[i]~dnorm(0,0.0001)
c[i]~dnorm(0,0.0001)
}

}#end model
```

Code in OpenBUGS-Effects of Biomass reduction treatments:

```
model{

for(i in 1:193){
```

```
ESmean[i]~dnorm(ESm[i],EStau[i])
ESm[i]<-a[Dist[i]]+SRE[study[i],res[i]]

}

for(r in 1:6){
for(i in 2:157){ SRE[i,r]~dnorm(0,T) }  }
T<-1/V
V~dunif(0,10000)

for(d in 1:8){
a[d]~dnorm(0,0.0001)}

}#end model
```

Code in OpenBUGS-Effects of disturbance severity:

```
model{

for(i in 1:204){

ESmean[i]~dnorm(ESm[i],EStau[i])
ESm[i]<-b[veg[i]]*TS[i]+(SRE[study[i]]-A)

}

for(i in 3:155){ SRE[i]~dnorm(A,T) }

T<-1/V
V~dunif(0,10000)
A~dnorm(0,0.0001)

for(i in 1:14){
b[i]~dnorm(0,0.0001)
}

}
```
