# Supplementary material for: Forest resilience under global environmental change: Do we have the information we need? A systematic review
Source: PLoS One. 2019 Sep 12;14(9):e0222207. doi: 10.1371/journal.pone.0222207 (PMC6742408; doi:10.1371/journal.pone.0222207)
Supplement: S3 Text — Publications included in the final analyses. (PDF) [file pone.0222207.s003.pdf]

Supporting information S3 Text

List of studies included in the analysis.

Abella, S. R., and P. J. Fornwalt. 2015. Ten years of vegetation assembly after a North American mega fire. Global Change Biology **21**:789-802.

Adamek, M., V. Hadincova, and J. Wild. 2016. Long-term effect of wildfires on temperate Pinus sylvestris forests: Vegetation dynamics and ecosystem resilience. Forest Ecology and Management **380**:285-295.

Alfaro-Sanchez, R., J. J. Camarero, F. R. Lopez-Serrano, R. Sanchez-Salguero, D. Moya, and J. De Las Heras. 2015. Positive coupling between growth and reproduction in young post-fire Aleppo pines depends on climate and site conditions. International Journal of Wildland Fire **24**:507-517.

Alfaro-Sanchez, R., R. Sanchez-Salguero, J. De las Heras, E. Hernandez-Tecles, D. Moya, and F. R. Lopez-Serrano. 2015. Vegetation dynamics of managed Mediterranean forests 16 yr after large fires in southeastern Spain. Applied Vegetation Science **18**:272-282.

Aung, T. T., Y. Mochida, and M. M. Than. 2013. Prediction of recovery pathways of cyclone-disturbed mangroves in the mega delta of Myanmar. Forest Ecology and Management **293**:103-113.

Baldwin, L. K., and G. E. Bradfield. 2010. Resilience of bryophyte communities in regenerating matrix forests after logging in temperate rainforests of coastal British Columbia. Botany-Botanique **88**:297-314.

Barlow, J., and C. A. Peres. 2008. Fire-mediated dieback and compositional cascade in an Amazonian forest. Philosophical Transactions of the Royal Society B-Biological Sciences **363**:1787-1794.

Bee, J. N., G. Kunstler, and D. A. Coomes. 2007. Resistance and resilience of New Zealand tree species to browsing. Journal of Ecology **95**:1014-1026.

Belien, E., S. Rossi, H. Morin, and A. Deslauriers. 2014. High-resolution analysis of stem radius variations in black spruce *Picea mariana* (Mill.) BSP subjected to rain exclusion for three summers. Trees-Structure and Function **28**:1257-1265.

Belote, R. T., R. H. Jones, and T. F. Wieboldt. 2012. Compositional stability and diversity of vascular plant communities following logging disturbance in Appalachian forests. Ecological Applications **22**:502-516.

Bennett, L. T., M. J. Bruce, J. MacHunter, M. Kohout, M. A. Tanase, and C. Aponte. 2016. Mortality and recruitment of fire-tolerant eucalypts as influenced by wildfire severity and recent prescribed fire. Forest Ecology and Management **380**:107-117.

Bernhardt-Romermann, M., A. Gray, A. J. Vanbergen, L. Berges, A. Bohner, R. W. Brooker, L. De Bruyn, B. De Cinti, T. Dirnbock, U. Grandin, A. J. Hester, R. Kanka, S. Klotz, G. Loucougaray, L. Lundin, G. Matteucci, I. Meszaros, O. Viktor, E. Preda, B. Prevosto, J. Pykala, W. Schmidt, M. E. Taylor, A. Vadineanu, T. Waldmann, and J. Stadler. 2011. Functional traits and local environment predict vegetation responses to disturbance: a pan-European multi-site experiment. Journal of Ecology **99**:777-787.

Bonfil, C., P. Cortes, J. M. Espelta, and J. Retana. 2004. The role of disturbance in the co-existence of the evergreen *Quercus ilex* and the deciduous Quercus cerrioides. Journal of Vegetation Science **15**:423-430.

Bormann, B. T., R. L. Darbyshire, P. S. Homann, B. A. Morrissette, and S. N. Little. 2015. Managing early succession for biodiversity and long-term productivity of conifer forests in southwestern Oregon. Forest Ecology and Management **340**:114-125.

Bottero, A., A. W. D'Amato, B. J. Palik, C. C. Kern, J. B. Bradford, and S. S. Scherer. 2017. Influence of Repeated Prescribed Fire on Tree Growth and Morialily in *Pinus resinosa* Forests, Northern Minnesota. Forest Science **63**:94-100.

Buma, B., and C. A. Wessman. 2012. Differential species responses to compounded perturbations and implications for landscape heterogeneity and resilience. Forest Ecology and Management **266**:25-33.

Caldeira, M. C., X. Lecomte, T. S. David, J. G. Pinto, M. N. Bugalho, and C. Werner. 2015. Synergy of extreme drought and shrub invasion reduce ecosystem functioning and resilience in water-limited climates. Scientific Reports **5**:15110.

Carnwath, G., and C. Nelson. 2017. Effects of biotic and abiotic factors on resistance versus resilience of Douglas fir to drought. Plos One **12**. doi.org/10.1371/journal.pone.0185604

Castro, J., C. Puerta-Pinero, A. B. Leverkus, G. Moreno-Rueda, and A. Sanchez-Miranda. 2012. Post-fire salvage logging alters a key plant-animal interaction for forest regeneration. Ecosphere **3**. doi.org/10.1890/ES12-00089.1

Chambers, M. E., P. J. Fornwalt, S. L. Malone, and M. A. Battaglia. 2016. Patterns of conifer regeneration following high severity wildfire in ponderosa pine - dominated forests of the Colorado Front Range. Forest Ecology and Management **378**:57-67.

Chandler, J. R., S. Haeussler, E. H. Hamilton, M. Feller, G. Bradfield, and S. W. Simard. 2017. Twenty years of ecosystem response after clearcutting and slashburning in conifer forests of central British Columbia, Canada. Plos One 12. doi.org/10.1371/journal.pone.0172667

Clarke, P. J., K. J. E. Knox, R. A. Bradstock, C. Munoz-Robles, and L. Kumar. 2014. Vegetation, terrain and fire history shape the impact of extreme weather on fire severity and ecosystem response. Journal of Vegetation Science **25**:1033-1044.

Clason, A. J., S. E. Macdonald, and S. Haeussler. 2014. Forest response to cumulative disturbance and stress: Two decades of change in whitebark pine ecosystems of west-central British Columbia. Ecoscience **21**:174-185.

Collins, B. M., A. J. Das, J. J. Battles, D. L. Fry, K. D. Krasnow, and S. L. Stephens. 2014. Beyond reducing fire hazard: fuel treatment impacts on overstory tree survival. Ecological Applications **24**:1879-1886.

Curzon, M. T., A. W. D'Amato, and B. J. Palik. 2016. Bioenergy harvest impacts to biodiversity and resilience vary across aspen-dominated forest ecosystems in the Lake States region, USA. Applied Vegetation Science **19**:667-678.

Curzon, M. T., A. W. D'Amato, S. Fraver, B. J. Palik, A. Bottero, J. R. Foster, and K. E. Gleason. 2017. Harvesting influences functional identity and diversity over time in forests of the northeastern USA. Forest Ecology and Management **400**:93-99.

D'Amato, A. W., J. B. Bradford, S. Fraver, and B. J. Palik. 2013. Effects of thinning on drought vulnerability and climate response in north temperate forest ecosystems. Ecological Applications **23**:1735-1742.

Day, N. J., S. Corriere, and J. L. Baltzer. 2017. Annual dynamics and resilience in post-fire boreal understory vascular plant communities. Forest Ecology and Management **401**:264-272.

de Grandpre, L., and Y. Bergeron. 1997. Diversity and stability of understorey communities following disturbance in the southern boreal forest. Journal of Ecology **85**:777-784.

De las Heras, J., D. Moya, F. R. Lopez-Serrano, and E. Rubio. 2013. Carbon sequestration of naturally regenerated Aleppo pine stands in response to early thinning. New Forests **44**:457-470.

de-Dios-Garcia, J., M. Pardos, and R. Calama. 2015. Interannual variability in competitive effects in mixed and monospecific forests of Mediterranean stone pine. Forest Ecology and Management **358**:230-239.

DeClerck, F. A. J., M. G. Barbour, and J. O. Sawyer. 2006. Species richness and stand stability in conifer forests of the Sierra Nevada. Ecology **87**:2787-2799.

Diaconu, D., H. P. Kahle, and H. Spiecker. 2017. Thinning increases drought tolerance of European beech: a case study on two forested slopes on opposite sides of a valley. European Journal of Forest Research **136:**319-328.

Diaz-Delgado, R., F. Lloret, X. Pons, and J. Terradas. 2002. Satellite evidence of decreasing resilience in Mediterranean plant communities after recurrent wildfires. Ecology **83**:2293-2303.

Ding, Y., R. G. Zang, S. R. Liu, F. L. He, and S. G. Letcher. 2012. Recovery of woody plant diversity in tropical rain forests in southern China after logging and shifting cultivation. Biological Conservation **145**:225-233.

Dodson, E. K., and H. T. Root. 2013. Conifer regeneration following stand-replacing wildfire varies along an elevation gradient in a ponderosa pine forest, Oregon, USA. Forest Ecology and Management **302**:163-170.

Dodson, E. K., D. W. Peterson, and R. J. Harrod. 2008. Understory vegetation response to thinning and burning restoration treatments in dry conifer forests of the eastern Cascades, USA. Forest Ecology and Management **255**:3130-3140.

Donato, D. C., J. B. Fontaine, W. D. Robinson, J. B. Kauffman, and B. E. Law. 2009. Vegetation response to a short interval between high-severity wildfires in a mixed-evergreen forest. Journal of Ecology **97**:142-154.

Dwomoh, F. K., and M. C. Wimberly. 2017. Fire regimes and forest resilience: alternative vegetation states in the West African tropics. Landscape Ecology **32**:1849-1865.

Elmqvist, T., M. Wall, A. L. Berggren, L. Blix, A. Fritioff, and U. Rinman. 2002. Tropical forest reorganization after cyclone and fire disturbance in Samoa: Remnant trees as biological legacies. Conservation Ecology **5**: 10.

Feller, I. C., E. M. Dangremond, D. J. Devlin, C. E. Lovelock, C. E. Proffitt, and W. Rodriguez. 2015. Nutrient enrichment intensifies hurricane impact in scrub mangrove ecosystems in the Indian River Lagoon, Florida, USA. Ecology **96**:2960-2972.

Fensham, R. J., J. L. Silcock, and J. M. Dwyer. 2011. Plant species richness responses to grazing protection and degradation history in a low productivity landscape. Journal of Vegetation Science **22**:997-1008.

Flores, B. M., R. Fagoaga, B. W. Nelson, and M. Holmgren. 2016. Repeated fires trap Amazonian blackwater floodplains in an open vegetation state. Journal of Applied Ecology **53**:1597-1603.

Gavinet, J., A. Vilagrosa, E. Chirino, M. E. Granados, V. R. Vallejo, and B. Prevosto. 2015. Hardwood seedling establishment below Aleppo pine depends on thinning intensity in two Mediterranean sites. Annals of Forest Science **72**:999-1008.

Gazol, A., and J. J. Camarero. 2016. Functional diversity enhances silver fir growth resilience to an extreme drought. Journal of Ecology **104**:1063-1075.

George, J. P., M. Grabner, S. Karanitsch-Ackerl, K. Mayer, L. Weissenbacher, and S. Schueler. 2017. Genetic variation, phenotypic stability, and repeatability of drought response in European larch throughout 50 years in a common garden experiment. Tree Physiology **37**:33-46.

Gibson, C. M., M. R. Turetsky, K. Cottenie, E. S. Kane, G. Houle, and E. S. Kasischke. 2016. Variation in plant community composition and vegetation carbon pools a decade following a severe fire season in interior Alaska. Journal of Vegetation Science **27**:1187-1197.

Gill, N. S., D. Jarvis, T. T. Veblen, S. T. A. Pickett, and D. Kulakowski. 2017. Is initial post-disturbance regeneration indicative of longer-term trajectories? Ecosphere **8**. doi.org/10.1002/ecs2.1924

Gower, K., J. B. Fontaine, C. Birnbaum, and N. J. Enright. 2015. Sequential Disturbance Effects of Hailstorm and Fire on Vegetation in a Mediterranean-Type Ecosystem. Ecosystems **18**:1121-1134.

Guada, G., J. J. Camarero, R. Sanchez-Salguero, and R. M. N. Cerrillo. 2016. Limited Growth Recovery after Drought-Induced Forest Dieback in Very Defoliated Trees of Two Pine Species. Frontiers in Plant Science **7**. doi: 10.3389/fpls.2016.00418.

Guillemot, J., E. K. Klein, H. Davi, and F. Courbet. 2015. The effects of thinning intensity and tree size on the growth response to annual climate in *Cedrus atlantica*: a linear mixed modeling approach. Annals of Forest Science **72**:651-663.

Haeussler, S., P. Bartemucci, and L. Bedford. 2004. Succession and resilience in boreal mixedwood plant communities 15-16 years after silvicultural site preparation. Forest Ecology and Management **199**:349-370.

Halofsky, J. E., and D. E. Hibbs. 2009. Controls on early post-fire woody plant colonization in riparian areas. Forest Ecology and Management **258**:1350-1358.

Hammett, E. J., M. W. Ritchie, and J. P. Berrill. 2017. Resilience of California black oak experiencing frequent fire: regeneration following two large wildfires 12 years apart. Fire Ecology 13:91-103.

Han, J., Z. H. Shen, L. X. Ying, G. X. Li, and A. P. Chen. 2015. Early post-fire regeneration of a fire-prone subtropical mixed Yunnan pine forest in Southwest China: Effects of pre-fire vegetation, fire severity and topographic factors. Forest Ecology and Management **356**:31-40.

Jakovac, C. C., M. Pena-Claros, T. W. Kuyper, and F. Bongers. 2015. Loss of secondary-forest resilience by land-use intensification in the Amazon. Journal of Ecology **103**:67-77.

Jimenez, M. N., and F. B. Navarro. 2016. Thinning effects on litterfall remaining after 8 years and improved stand resilience in Aleppo pine afforestation (SE Spain). Journal of Environmental Management **169**:174-183.

Karlo, T., and N. Sajna. 2017. Biodiversity related understorey stability of small peri-urban forest after a 100-year recurrent flood. Landscape and Urban Planning **162**:104-114.

Keeling, E. G., A. Sala, and T. H. DeLuca. 2011. Lack of fire has limited physiological impact on old-growth ponderosa pine in dry montane forests of north-central Idaho. Ecological Applications **21**:3227-3237.

Kerhoulas, L. P., T. E. Kolb, M. D. Hurteau, and G. W. Koch. 2013. Managing climate change adaptation in forests: a case study from the US Southwest. Journal of Applied Ecology **50**:1311-1320.

Kern, C. C., A. W. D'Arnato, and T. F. Strong. 2013. Diversifying the composition and structure of managed, late-successional forests with harvest gaps: What is the optimal gap size? Forest Ecology and Management **304**:110-120.

Keyser, T. L., and P. M. Brown. 2014. Climate-growth relationships for yellow-poplar across structural and site quality gradients in the southern Appalachian Mountains. Forest Ecology and Management **329**:158-165.

Keyser, T. L., and P. M. Brown. 2016. Drought response of upland oak (Quercus L.) species in Appalachian hardwood forests of the southeastern USA. Annals of Forest Science **73**:971-986.

Knox, K. J. E., and P. J. Clarke. 2011. Fire severity and nutrient availability do not constrain resprouting in forest shrubs. Plant Ecology **212**:1967-1978.

Kohler, M., J. Sohn, G. Nagele, and J. Bauhus. 2010. Can drought tolerance of Norway spruce (Picea abies (L.) Karst.) be increased through thinning? European Journal of Forest Research **129**:1109-1118.

Kohv, K., M. Zobel, and J. Liira. 2013. The resilience of the forest field layer to anthropogenic disturbances depends on site productivity. Canadian Journal of Forest Research-Revue Canadienne De Recherche Forestiere **43**:1040-1049.

Kranabetter, J. M., S. Haeussler, and C. Wood. 2017. Vulnerability of boreal indicators (ground-dwelling beetles, understory plants and ectomycorrhizal fungi) to severe forest soil disturbance. Forest Ecology and Management **402**:213-222.

Krasnow, K. D., and S. L. Stephens. 2015. Evolving paradigms of aspen ecology and management: impacts of stand condition and fire severity on vegetation dynamics. Ecosphere **6**. doi.org/10.1890/ES14-00354.1

Kuss, F. R., and C. N. Hall. 1991. Ground flora trampling studies – 5 years after closure. Environmental Management **15**:715-727.

Larson, A. J., J. A. Lutz, R. F. Gersonde, J. F. Franklin, and F. F. Hietpas. 2008. Potential site productivity influences the rate of forest structural development. Ecological Applications **18**:899-910.

Leak, W. B., and M. L. Smith. 1996. Sixty years of management and natural disturbance in a New England forested landscape. Forest Ecology and Management **81**:63-73.

Levesque, M., K. P. McLaren, and M. A. McDonald. 2011. Recovery and dynamics of a primary tropical dry forest in Jamaica, 10 years after human disturbance. Forest Ecology and Management **262**:817-826.

Lewis, T., and V. J. Debuse. 2012. Resilience of a eucalypt forest woody understorey to long-term (34-55 years) repeated burning in subtropical Australia. International Journal of Wildland Fire **21**:980-991.

Liu, C. X., P. A. Harcombe, and R. G. Knox. 2004. Structural changes after prescribed fire in woody plant communities of southeastern Texas. Texas Journal of Science **56**:319-334.

Lloret, F., and C. Garcia. 2016. Inbreeding and neighbouring vegetation drive drought-induced die-off within juniper populations. Functional Ecology **30**:1696-1704.

Lloret, F., D. Siscart, and C. Dalmases. 2004. Canopy recovery after drought dieback in holm-oak Mediterranean forests of Catalonia (NE Spain). Global Change Biology **10**:2092-2099.

Lloret, F., E. G. Keeling, and A. Sala. 2011. Components of tree resilience: effects of successive low-growth episodes in old ponderosa pine forests. Oikos **120**:1909-1920.

Lopez, B. C., C. A. Gracia, S. Sabate, and T. Keenan. 2009. Assessing the resilience of Mediterranean holm oaks to disturbances using selective thinning. Acta Oecologica-International Journal of Ecology **35**:849-854.

Macamo, C. C. F., E. Massuanganhe, D. K. Nicolau, S. O. Bandeira, and J. B. Adams. 2016. Mangrove's response to cyclone Eline (2000): What is happening 14 years later. Aquatic Botany **134**:10-17.

Magruder, M., S. Chhin, A. Monks, and J. O'Brien. 2012. Effects of Initial Stand Density and Climate on Red Pine Productivity within Huron National Forest, Michigan, USA. Forests **3**:1086-1103.

Magruder, M., S. Chhin, B. Palik, and J. B. Bradford. 2013. Thinning increases climatic resilience of red pine. Canadian Journal of Forest Research **43**:878-889.

Malak, D. A., and J. G. Pausas. 2006. Fire regime and post-fire Normalized Difference Vegetation Index changes in the eastern Iberian peninsula (Mediterranean basin). International Journal of Wildland Fire **15**:407-413.

Mansuy, N., S. Gauthier, A. Robitaille, and Y. Bergeron. 2012. Regional patterns of postfire canopy recovery in the northern boreal forest of Quebec: interactions between surficial deposit, climate, and fire cycle. Canadian Journal of Forest Research-Revue Canadienne De Recherche Forestiere **42**:1328-1343.

Maringer, J., M. Conedera, D. Ascoli, D. R. Schmatz, and T. Wohlgemuth. 2016. Resilience of European beech forests (*Fagus sylvatica* L.) after fire in a global change context. International Journal of Wildland Fire **25**:699-710.

Marques, L., J. J. Camarero, A. Gazol, and M. A. Zavala. 2016. Drought impacts on tree growth of two pine species along an altitudinal gradient and their use as early-warning signals of potential shifts in tree species distributions. Forest Ecology and Management **381**:157-167.

Marra, D. M., J. Q. Chambers, N. Higuchi, S. E. Trumbore, G. Ribeiro, J. dos Santos, R. I. Negron-Juarez, B. Reu, and C. Wirth. 2014. Large-Scale Wind Disturbances Promote Tree Diversity in a Central Amazon Forest. Plos One **9**. doi.org/10.1371/journal.pone.0103711

Martins, K. G., M. C. M. Marques, E. dos Santos, and R. Marques. 2015. Effects of soil conditions on the diversity of tropical forests across a successional gradient. Forest Ecology and Management **349**:4-11.

McNamara, S., P. D. Erskine, D. Lamb, L. Chantalangsy, and S. Boyle. 2012. Primary tree species diversity in secondary fallow forests of Laos. Forest Ecology and Management **281**:93-99.

Merlin, M., T. Perot, S. Perret, N. Korboulewsky, and P. Vallet. 2015. Effects of stand composition and tree size on resistance and resilience to drought in sessile oak and Scots pine. Forest Ecology and Management **339**:22-33.

Minor, J., D. A. Falk, and G. A. Barron-Gafford. 2017. Fire Severity and Regeneration Strategy Influence Shrub Patch Size and Structure Following Disturbance. Forests **8**. doi.org/10.3390/f8070221

Moris, J. V., G. Vacchiano, S. R. Enri, M. Lonati, R. Motta, and D. Ascoli. 2017. Resilience of European larch (*Larix decidua* Mill.) forests to wildfires in the western Alps. New Forests **48**:663-683.

Morrissey, R. C., M. R. Saunders, and M. A. Jenkins. 2015. Successional and structural responses to overstorey disturbance in managed and unmanaged forests. Forestry **88**:376-389.

Moya, D., J. D. L. Heras, F. R. Lopez-Serrano, and V. Leone. 2008. Optimal intensity and age of management in young Aleppo pine stands for post-fire resilience. Forest Ecology and Management **255**:3270-3280.

Moya, D., J. de las Heras, F. R. Lopez-Serrano, and P. Ferrandis. 2015. Post-Fire Seedling Recruitment and Morpho-Ecophysiological Responses to Induced Drought and Salvage Logging in Pinus halepensis Mill. Stands. Forests **6**:1858-1877.

Mwavu, E. N., and E. T. F. Witkowski. 2009. Seedling regeneration, environment and management in a semi-deciduous African tropical rain forest. Journal of Vegetation Science **20**:791-804.

Nino, M., K. P. McLaren, H. Meilby, M. Levesque, B. Wilson, and M. McDonald. 2014. Long-term changes in above ground biomass after disturbance in a neotropical dry forest, Hellshire Hills, Jamaica. Plant Ecology **215**:1081-1097.

Nishimua, T. B., E. Suzuki, T. Kohyama, and S. Tsuyuzaki. 2007. Mortality and growth of trees in peat-swamp and heath forests in Central Kalimantan after severe drought. Plant Ecology **188**:165-177.

Parro, K., M. Metslaid, G. Renel, A. Sims, J. A. Stanturf, K. Jogiste, and K. Koster. 2015. Impact of postfire management on forest regeneration in a managed hemiboreal forest, Estonia. Canadian Journal of Forest Research **45**:1192-1197.

Pettit, N. E., and R. J. Naiman. 2005. Flood-deposited wood debris and its contribution to heterogeneity and regeneration in a semi-arid riparian landscape. Oecologia **145**:434-444.

Pettit, N. E., and R. J. Naiman. 2007. Postfire response of flood-regenerating riparian vegetation in a semi-arid landscape. Ecology **88**:2094-2104.

Poorter, L., F. B. Ongers, T. M. Aide, A. M. Almeyda Zambrano, P. Balvanera, J. M. Becknell, V. Boukili, P. H. S. Brancalion, E. N. Broadbent, R. L. Chazdon, D. Craven, J. S. de Almeida-Cortez, G. A. L. Cabral, B. H. J. de Jong, J. S. Denslow, D. H. Dent, S. J. DeWalt, J. M. Dupuy, S. M. Duran, M. M. Espirito-Santo, M. C. Fandino, R. G. Cesar, J. S. Hall, J. L. Hernandez-Stefanoni, C. C. Jakovac, A. B. Junqueira, D. Kennard, S. G. Letcher, J. C. Licona, M. Lohbeck, E. Marin-Spiotta, M. Martinez-Ramos, P. Massoca, J. A. Meave, R. Mesquita, F. Mora, R. Munoz, R. Muscarella, Y. R. F. Nunes, S. Ochoa-Gaona, A. A. de Oliveira, E. Orihuela-Belmonte, M. Pena-Claros, E. A. Perez-Garcia, D. Piotto, J. S. Powers, J. Rodriguez-Velazquez, I. E. Romero-Perez, J. Ruiz, J. G. Saldarriaga, A. Sanchez-Azofeifa, N. B. Schwartz, M. K. Steininger, N. G. Swenson, M. Toledo, M. Uriarte, M. van Breugel, H. van der Wal, M. D. M. Veloso, H. F. M. Vester, A. Vicentini, I. C. G. Vieira, T. V. Bentos, G. B. Williamson, and D. M. A. Rozendaal. 2016. Biomass resilience of Neotropical secondary forests. Nature **530**:211-214.

Powers, E. M., J. D. Marshall, J. W. Zhang, and L. Wei. 2013. Post-fire management regimes affect carbon sequestration and storage in a Sierra Nevada mixed conifer forest. Forest Ecology and Management **291**:268-277.

Prabakaran, N., and B. Paramasivam. 2014. Recovery rate of vegetation in the tsunami impacted littoral forest of Nicobar Islands, India. Forest Ecology and Management **313**:243-253.

Premer, M. I., R. E. Froese, C. R. Webster, and L. M. Nagel. 2016. Vegetation response to logging residue removals in Great Lakes aspen forests: Long-term trends under operational management. Forest Ecology and Management **382**:257-268.

Pretzsch, H., G. Schutze, and E. Uhl. 2013. Resistance of European tree species to drought stress in mixed versus pure forests: evidence of stress release by inter-specific facilitation. Plant Biology **15**:483-495.

Raffaele, E., T. T. Veblen, M. Blackhall, and N. Tercero-Bucardo. 2011. Synergistic influences of introduced herbivores and fire on vegetation change in northern Patagonia, Argentina. Journal of Vegetation Science **22**:59-71.

Rais, A., J. W. G. van de Kuilen, and H. Pretzsch. 2014. Growth reaction patterns of tree height, diameter, and volume of Douglas-fir (*Pseudotsuga menziesii* Mirb. Franco) under acute drought stress in Southern Germany. European Journal of Forest Research **133**:1043-1056.

Reinikainen, M., A. W. D'Amato, J. B. Bradford, and S. Fraver. 2014. Influence of stocking, site quality, stand age, low-severity canopy disturbance, and forest composition on sub-boreal aspen mixedwood carbon stocks. Canadian Journal of Forest Research-Revue Canadienne De Recherche Forestiere **44**:230-242.

Ristau, T. E., S. H. Stoleson, S. B. Horsley, and D. S. deCalesta. 2011. Ten-year response of the herbaceous layer to an operational herbicide-shelterwood treatment in a northern hardwood forest. Forest Ecology and Management **262**:970-979.

Romme, W. H., T. G. Whitby, D. B. Tinker, and M. G. Turner. 2016. Deterministic and stochastic processes lead to divergence in plant communities 25 years after the 1988 Yellowstone fires. Ecological Monographs **86**:327-351.

Rother, M. T., and T. T. Veblen. 2016. Limited conifer regeneration following wildfires in dry ponderosa pine forests of the Colorado Front Range. Ecosphere **7**. doi.org/10.1002/ecs2.1594

Rydgren, K., R. H. Okland, and G. Hestmark. 2004. Disturbance severity and community resilience in a boreal forest. Ecology **85**:1906-1915.

Sampaio, A. B. 2007. Regeneration of seasonal deciduous forest tree species in long-used pastures in central Brazil. Biotropica **39**:655-659.

Sansevero, J. B. B., P. V. Prieto, A. Sanchez-Tapia, J. M. A. Braga, and P. Rodrigues. 2017. Past land-use and ecological resilience in a lowland Brazilian Atlantic Forest: implications for passive restoration. New Forests **48**:573-586.

Schafer, C., T. E. E. Grams, T. Rotzer, A. Feldermann, and H. Pretzsch. 2017. Drought Stress Reaction of Growth and Delta C-13 in Tree Rings of European Beech and Norway Spruce in Monospecific Versus Mixed Stands Along a Precipitation Gradient. Forests **8**. doi.org/10.3390/f8060177

Schmalholz, M., and K. Hylander. 2011. Boulders increase resistance to clear-cut logging but not subsequent recolonization rates of boreal bryophytes. Oecologia **167**:1093-1101.

Seidl, R., F. Vigl, G. Rossler, M. Neumann, and W. Rammer. 2017. Assessing the resilience of Norway spruce forests through a model-based reanalysis of thinning trials. Forest Ecology and Management **388**:3-12.

Shields, J. M., and C. R. Webster. 2007. Ground-layer response to group selection with legacy-tree retention in a managed northern hardwood forest. Canadian Journal of Forest Research-Revue Canadienne De Recherche Forestiere **37**:1797-1807.

Stevens, J. T., H. D. Safford, and A. M. Latimer. 2014. Wildfire-contingent effects of fuel treatments can promote ecological resilience in seasonally dry conifer forests. Canadian Journal of Forest Research-Revue Canadienne De Recherche Forestiere **44**:843-854.

Strengbom, J., and A. Nordin. 2008. Commercial forest fertilization causes long-term residual effects in ground vegetation of boreal forests. Forest Ecology and Management **256**:2175-2181.

Stuart-Haentjens, E. J., P. S. Curtis, R. T. Fahey, C. S. Vogel, and C. M. Gough. 2015. Net primary production of a temperate deciduous forest exhibits a threshold response to increasing disturbance severity. Ecology **96**:2478-2487.

Taeger, S., C. Zang, M. Liesebach, V. Schneck, and A. Menzel. 2013. Impact of climate and drought events on the growth of Scots pine (Pinus sylvestris L.) provenances. Forest Ecology and Management **307**:30-42.

Temperli, C., S. J. Hart, T. T. Veblen, D. Kulakowski, J. J. Hicks, and R. Andrus. 2014. Are density reduction treatments effective at managing for resistance or resilience to spruce beetle disturbance in the southern Rocky Mountains? Forest Ecology and Management **334**:53-63.

Tepley, A. J., J. R. Thompson, H. E. Epstein, and K. J. Anderson-Teixeira. 2017. Vulnerability to forest loss through altered postfire recovery dynamics in a warming climate in the Klamath Mountains. Global Change Biology **23**:4117-4132.

Tessler, N., L. Wittenberg, E. Provizor, and N. Greenbaum. 2014. The influence of short-interval recurrent forest fires on the abundance of Aleppo pine (Pinus halepensis Mill.) on Mount Carmel, Israel. Forest Ecology and Management **324**:109-116.

Trouve, R., J. D. Bontemps, C. Collet, I. Seynave, and F. Lebourgeois. 2017. Radial growth resilience of sessile oak after drought is affected by site water status, stand density, and social status. Trees-Structure and Function **31**:517-529.

Trubina, M. R. 2009. Species richness and resilience of forest communities: combined effects of short-term disturbance and long-term pollution. Plant Ecology **201**:339-350.

Tsitsoni, T., P. Ganatsas, T. Zagas, and A. Tsakaldimi. 2004. Dynamics of postfire regeneration of *Pinus brutia* Ten. in an artificial forest ecosystem of northern Greece. Plant Ecology **171**:165-174.

Urli, M., N. Thiffault, M. Barrette, L. Belanger, A. Leduc, and D. Chalifour. 2017. Key ecosystem attributes and productivity of boreal stands 20 years after the onset of silviculture scenarios of increasing intensity. Forest Ecology and Management **389**:404-416.

van Lent, J., J. C. Hernandez-Barrios, N. P. R. Anten, and M. Martinez-Ramos. 2014. Defoliation effects on seed dispersal and seedling recruitment in a tropical rain forest understorey palm. Journal of Ecology **102**:709-720.

Vanha-Majamaa, I., E. Shorohova, H. Kushnevskaya, and J. Jalonen. 2017. Resilience of understory vegetation after variable retention felling in boreal Norway spruce forests - A ten-year perspective. Forest Ecology and Management **393**:12-28.

Vega, S. G. D., J. D. Heras, and D. Moya. 2016. Resilience of Mediterranean terrestrial ecosystems and fire severity in semiarid areas: Responses of Aleppo pine forests in the short, mid and long term. Science of the Total Environment **573**:1171-1177.

Verbesselt, J., N. Umlauf, M. Hirota, M. Holmgren, E. H. Van Nes, M. Herold, A. Zeileis, and M. Scheffer. 2016. Remotely sensed resilience of tropical forests. Nature Climate Change **6**:1028-1031.

Vicente-Serrano, S. M., F. Perez-Cabello, and T. Lasanta. 2011. *Pinus halepensis* regeneration after a wildfire in a semiarid environment: assessment using multitemporal Landsat images. International Journal of Wildland Fire **20**:195-208.

von Oheimb, G., W. Hardtle, D. Eckstein, H. H. Engelke, T. Hehnke, B. Wagner, and A. Fichtner. 2014. Does Forest Continuity Enhance the Resilience of Trees to Environmental Change? Plos One **9**. doi.org/10.1371/journal.pone.0113507

Walker, X. J., M. C. Mack, and J. F. Johnstone. 2017. Predicting Ecosystem Resilience to Fire from Tree Ring Analysis in Black Spruce Forests. Ecosystems **20**:1137-1150.

Waltz, A. E. M., M. T. Stoddard, E. L. Kalies, J. D. Springer, D. W. Huffman, and A. S. Meador. 2014. Effectiveness of fuel reduction treatments: Assessing metrics of forest resiliency and wildfire severity after the Wallow Fire, AZ. Forest Ecology and Management **334**:43-52.

Welch, K. R., H. D. Safford, and T. P. Young. 2016. Predicting conifer establishment post wildfire in mixed conifer forests of the North American Mediterranean-climate zone. Ecosphere **7**. doi.org/10.1002/ecs2.1609

Wiechmann, M. L., M. D. Hurteau, M. P. North, G. W. Koch, and L. Jerabkova. 2015. The carbon balance of reducing wildfire risk and restoring process: an analysis of 10-year post-treatment carbon dynamics in a mixed-conifer forest. Climatic Change **132**:709-719.

Windmuller-Campione, M. A., and J. N. Long. 2015. If Long-Term Resistance to a Spruce Beetle Epidemic is Futile, Can Silvicultural Treatments Increase Resilience in Spruce-Fir Forests in the Central Rocky Mountains? Forests **6**:1157-1178.

Wolf, J. H. D. 2005. The response of epiphytes to anthropogenic disturbance of pine-oak forests in the highlands of Chiapas, Mexico. Forest Ecology and Management **212**:376-393.

Xu, C. Y., H. Y. Liu, O. A. Anenkhonov, A. Y. Korolyuk, D. V. Sandanov, L. D. Balsanova, B. B. Naidanov, and X. C. Wu. 2017. Long-term forest resilience to climate change indicated by mortality, regeneration, and growth in semiarid southern Siberia. Global Change Biology **23**:2370-2382.

Zang, C., C. Hartl-Meier, C. Dittmar, A. Rothe, and A. Menzel. 2014. Patterns of drought tolerance in major European temperate forest trees: climatic drivers and levels of variability. Global Change Biology **20**:3767-3779.

Zenner, E. K., Y. L. Dickinson, and J. E. Peck. 2013. Recovery of forest structure and composition to harvesting in different strata of mixed even-aged central Appalachian hardwoods. Annals of Forest Science **70**:151-159.

Zuleta, D., A. Duque, D. Cardenas, H. C. Muller-Landau, and S. J. Davies. 2017. Drought-induced mortality patterns and rapid biomass recovery in a terra firme forest in the Colombian Amazon. Ecology **98**:2538-2546.
