# Supplementary material for: Forest resilience under global environmental change: Do we have the information we need? A systematic review
Source: PLoS One. 2019 Sep 12;14(9):e0222207. doi: 10.1371/journal.pone.0222207 (PMC6742408; doi:10.1371/journal.pone.0222207)
Supplement: S1 Fig — a) Proportion of the data under each disturbance for the two systems. b) Proportion of the data under each disturbance and context combination for the two systems. Numbers in parenthesis indicated number of observations. (PDF) [file pone.0222207.s005.pdf]

Supplemental information S1 Fig

Distribution of observations

S1 Fig a) Proportion of the data under each disturbance for the two systems. b) Proportion of the data under each disturbance and context combination for the two systems. Numbers in parenthesis indicated number of observations.

a)

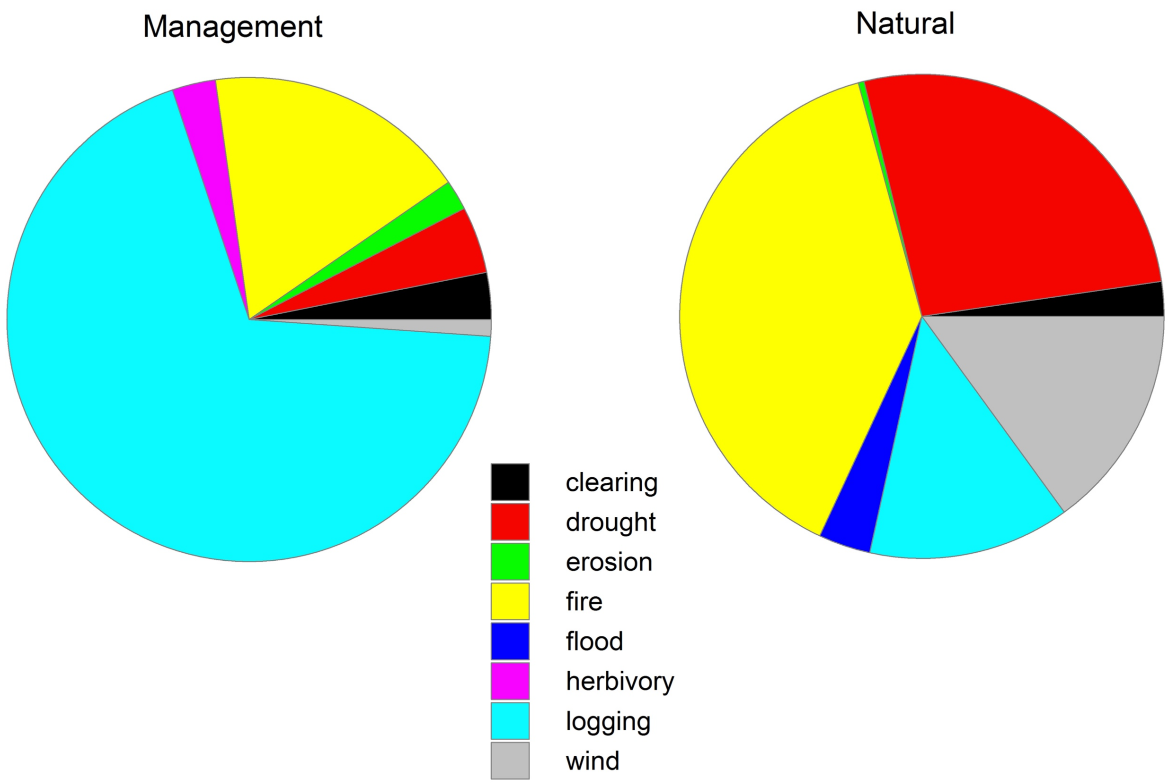

b)

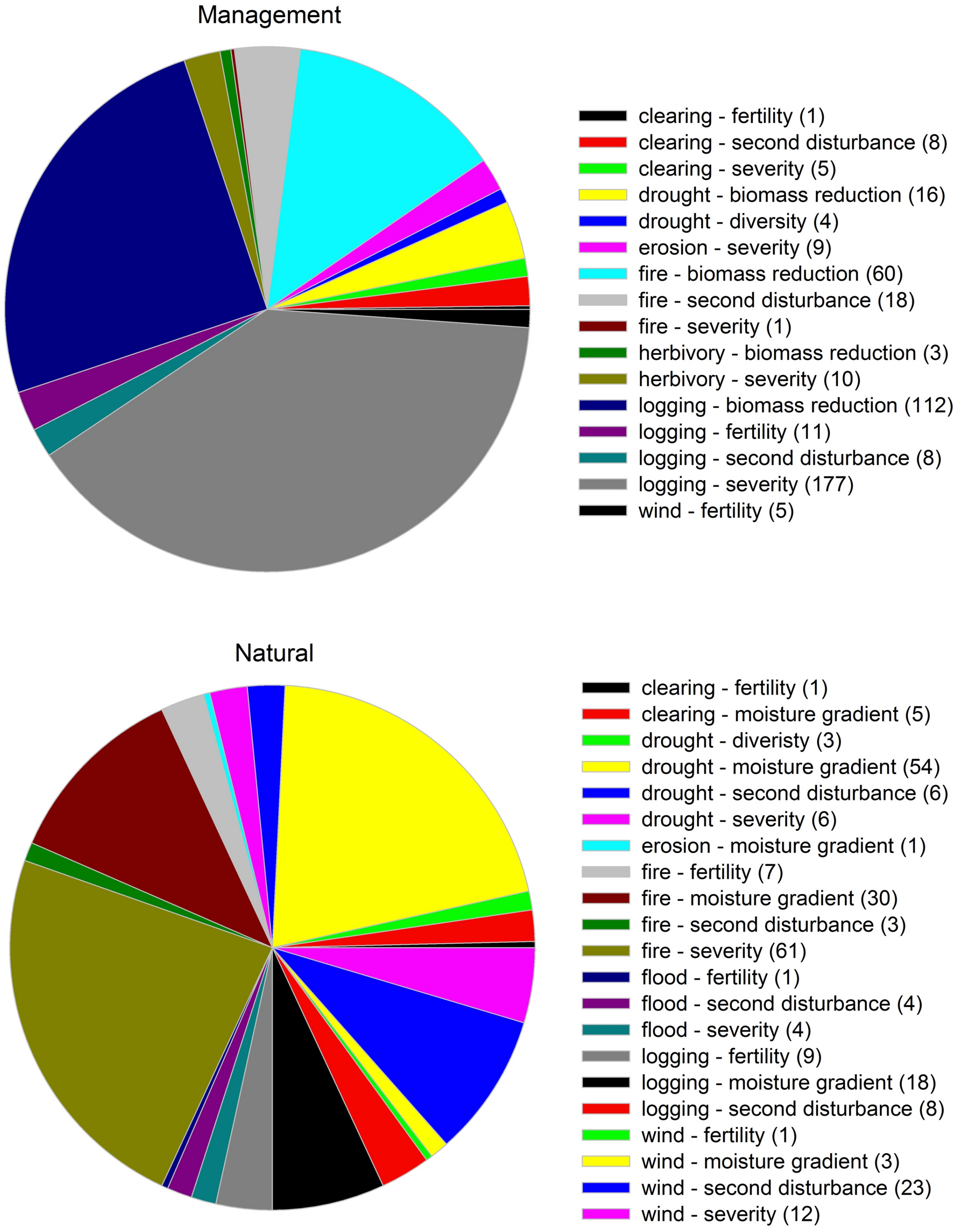
Management
clearing - fertility (1)
clearing - second disturbance (8)
clearing - severity (5)
drought - biomass reduction (16)
drought - diversity (4)
erosion - severity (9)
fire - biomass reduction (60)
fire - second disturbance (18)
fire - severity (1)
herbivory - biomass reduction (3)
herbivory - severity (10)
logging - biomass reduction (112)
logging - fertility (11)
logging - second disturbance (8)
logging - severity (177)
wind - fertility (5)
Natural
clearing - fertility (1)
clearing - moisture gradient (5)
drought - diveristy (3)
drought - moisture gradient (54)
drought - second disturbance (6)
drought - severity (6)
erosion - moisture gradient (1)
fire - fertility (7)
fire - moisture gradient (30)
fire - second disturbance (3)
fire - severity (61)
flood - fertility (1)
flood - second disturbance (4)
flood - severity (4)
logging - fertility (9)
logging - moisture gradient (18)
logging - second disturbance (8)
wind - fertility (1)
wind - moisture gradient (3)
wind - second disturbance (23)
wind - severity (12)
