# Supplementary material for: Forest resilience under global environmental change: Do we have the information we need? A systematic review
Source: PLoS One. 2019 Sep 12;14(9):e0222207. doi: 10.1371/journal.pone.0222207 (PMC6742408; doi:10.1371/journal.pone.0222207)
Supplement: S2 Fig — (PDF) [file pone.0222207.s006.pdf]

Supplemental materials S2 Fig

PRISMA 2009 Flow Diagram

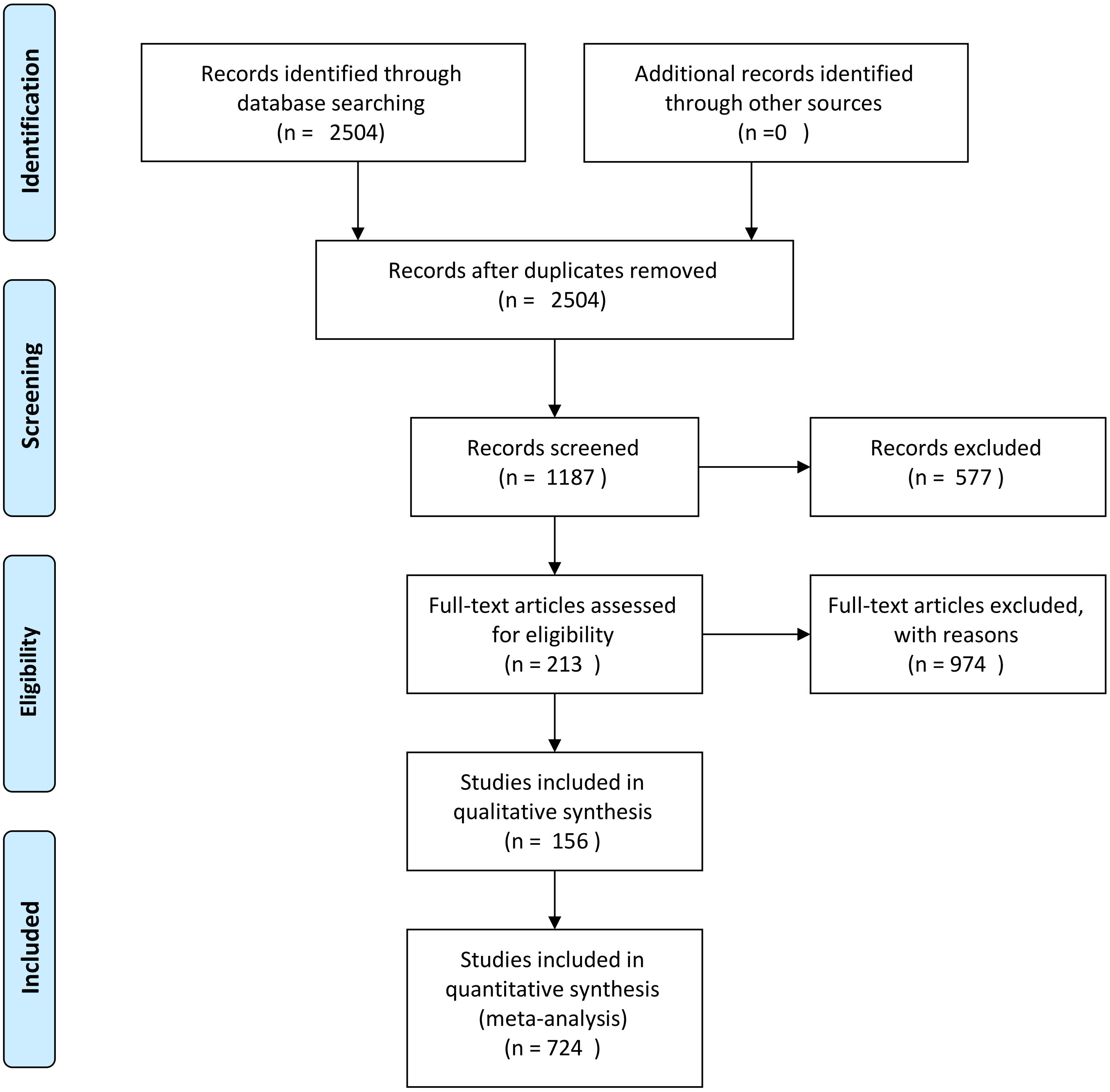

From: Moher D, Liberati A, Tetzlaff J, Altman DG, The PRISMA Group (2009). *P*referred *R*eporting *I*tems for *S*ystematic Reviews and *M*eta-*A*nalyses: The PRISMA Statement. PLoS Med 6(7): e1000097. doi:10.1371/journal.pmed1000097

For more information, visit www.prisma-statement.org.
