# Supplementary material for: Forest resilience under global environmental change: Do we have the information we need? A systematic review
Source: PLoS One. 2019 Sep 12;14(9):e0222207. doi: 10.1371/journal.pone.0222207 (PMC6742408; doi:10.1371/journal.pone.0222207)
Supplement: S1 Table — Numbers in bold indicate statistically significant effect sizes or covariates, different letters indicate statistically significant differences between biomes or strata (i.e., 95% credible intervals [CI] do not intersect with zero). (PDF) [file pone.0222207.s007.pdf]

Supplemental information S1 Table

<u>Parameter values, means, SD and 95%CI.</u>

S1 Table. Numbers in bold indicate statistically significant effect sizes or covariates, different letters indicate statistically significant differences between biomes or strata (i.e., 95% credible intervals [CI] do not intersect with zero).

Parameter	Mean±SD	95%CI
**Hierarchical analysis of effect sizes**		
$ES1_{managed,biomass\ reduction,\ abundance}$	0.114±0.198	-0.194, 0.508
$ES1_{managed,biomass\ reduction,\ change}$	0.442±0.963	-1.50, 2.29
$ES1_{managed,biomass\ reduction,\ diversity}$	0.324±0.322	-0.183, 1.15
$ES1_{managed,biomass\ reduction,\ growth}$	0.207±0.165	-0.088, 0.483
$ES1_{managed,biomass\ reduction,\ reproduction}$	**3.04±0.188**	**2.69, 3.4**
$ES1_{managed,biomass\ reduction,\ resilience}$	-0.049±0.616	-1.27, 0.993
$ES2_{managed,biomass\ reduction}$	0.649±0.755	-0.919, 2.14
$\sigma^2_{managed,\ biomass\ reduction}$	3.46±2.23	0.765, 9.0
$ES1_{managed,diveristy,\ growth}$	0.074±0.931	-1.80, 1.91
$ES1_{managed,diveristy,\ resilience}$	0.041±0.998	-2.71, 2.74
$ES2_{managed,\ diversity}$	0.050±1.37	-2.74, 2.85
$\sigma^2_{managed,\ diversity}$	3.96±2.87	0.114, 9.57
$ES1_{natural,diveristy,\ diversity}$	0.022±3.89	-7.61, 7.71
$ES1_{natural,\ diversity,\ growth}$	0.016±3.88	-7.58, 7.71
$ES1_{natural,\ diversity,\ resilience}$	0.028±3.89	-7.58, 7.58
$ES2_{natural,\ diversity}$	-0.094±1.14	-2.41, 2.20
$\sigma^2_{natural,\ diversity}$	3.86±2.77	0.151, 9.51
$ES1_{managed,\ fertility,\ abundance}$	-0.210±0.949	-2.12, 1.58
$ES1_{managed,\ fertility,\ growth}$	0.217±0.705	-1.06, 1.66
$ES2_{managed,\ fertility}$	0.007±1.33	-2.71, 2.74
$\sigma^2_{managed,\ fertility}$	3.90±2.87	0.101, 9.57
$ES1_{natural,\ fertility,\ abundance}$	0.484±0.63	-0.657, 1.71
$ES1_{natural,\ fertility,\ diversity}$	0.531±0.52	-0.491, 1.55
$ES1_{natural,\ fertility,\ growth}$	0.582±0.597	-0.586, 1.70
$ES1_{natural,\ fertility,\ resilience}$	0.238±0.585	-0.844, 1.31
$ES2_{natural,\ fertility}$	0.441±0.722	-1.08, 1.93
$\sigma^2_{natural,\ fertility}$	1.93±2.23	0.040, 8. 46
$ES1_{natural,\ moisture\ gradient,\ abundance}$	**0.533±0.225**	**0.143, 0.960**
$ES1_{natural,\ moisture\ gradient,\ diversity}$	**0.685±0.353**	**0.144, 1.71**
$ES1_{natural,\ moisture\ gradient,\ growth}$	0.205±0.287	-0.514, 0.661
$ES1_{natural,\ moisture\ gradient,\ reproduction}$	**2.85±0.15**	**2.61,3.14**
$ES1_{natural,\ moisture\ gradient,\ resilience}$	-0.129±0.071	-0.246, 0.034

$ES2_{natural, moisture\ gradient}$	0.782±0.801	-0.877, 2.39
$\sigma^2_{natural, moisture\ gradient}$	3.54±2.34	0.706, 9.15
$ES1_{managed, second\ disturbance, abundance}$	-0.490±0.6	-1.69, 0.54
$ES1_{managed, second\ disturbance, change}$	-0.516±0.73	-2.10, 0.880
$ES1_{managed, second\ disturbance, diversity}$	-0.439±0.588	-1.61, 0.735
$ES1_{managed, second\ disturbance, growth}$	-0.393±0.517	-1.43, 0.532
$ES2_{managed, second\ disturbance}$	-0.444±0.733	-1.95, 1.03
$\sigma^2_{managed, second\ disturbance}$	1.68±2.12	0.022, 8.13
$ES1_{natural, second\ disturbance, abundance}$	-0.024±0.246	-0.479, 0.442
$ES1_{natural, second\ disturbance, diversity}$	-0.286±0.337	-0.973, 0.352
$ES1_{natural, second\ disturbance, growth}$	0.112±0.363	-0.534, 0.787
$ES1_{natural, second\ disturbance, resilience}$	**-0.706±0.505**	**-1.86, -0.007**
$ES2_{natural, second\ disturbance}$	-0.215±0.627	-1.58, 1.15
$\sigma^2_{natural, second\ disturbance}$	1.65±2.16	0.017, 8.21
$ES1_{managed, severity, abundance}$	**1.88±0.002**	**1.87, 1.88**
$ES1_{managed, severity, change}$	-0.284±0.656	-1.48, 1.18
$ES1_{managed, severity, diversity}$	-0.128±0.178	-0.458, 0.213
$ES1_{managed, severity, growth}$	-0.192±0.332	-0.702, 0.371
$ES1_{managed, severity, reproduction}$	0.196±0.96	-1.62, 2.09
$ES1_{managed, severity, resilience}$	0.621±0.747	-0.573, 1.99
$ES2_{managed, severity}$	0.340±0.681	-1.06, 1.70
$\sigma^2_{managed, severity}$	2.54±2.04	0.422, 8.29
$ES1_{natural, severity, abundance}$	-0.235±0.228	-0.598, 0.221
$ES1_{natural, severity, diversity}$	0.145±0.336	-0.541, 0.783
$ES1_{natural, severity, growth}$	**1.62±0.409**	**1.06, 2.34**
$ES1_{natural, severity, resilience}$	-0.077±0.853	-1.78, 1.55
$ES2_{natural, severity}$	0.340±0.88	-1.49, 2.12
$\sigma^2_{natural, severity}$	3.18±2.48	0.338, 9.13
$\sigma^2_{study\ random\ effects}$	1.06±0.138	0.833, 1.37
**Analysis of biomass reduction treatments**		
$\alpha_{drought}$	**0.440±0.171**	**0.090, 0.771**
α_{fire}	-0.007±0.092	-0.225, 0.152
$\alpha_{herbivory}$	0.776±0.492	-0.195, 1.74
$\alpha_{logging}$	0.113±0.093	-0.104, 0.273
$\sigma\alpha^2_{study\ random\ effects}$	0.465±0.110	0.296, 0.724
**Analysis of Moisture gradients**		
intercept $\beta 1_{boreal}$	1.39±0.312	0.052, 2.59 a
intercept $\beta 1_{Mediterranean}$	0.491±0.431	-0.425, 1.36 a
intercept $\beta 1_{temperate}$	0.205±0.148	-0.026, 0.498 a
intercept $\beta 1_{tropical}$	-0.209±0.704	-1.53, 1.21 a
effect of location DMI $\beta 2_{boreal}$	**1.44±0.206**	**1.02, 1.84 a**
effect of location DMI $\beta 2_{Mediterranean}$	0.310±0.438	-0.465, 1.19 ab
effect of location DMI $\beta 2_{temperate}$	-0.326±0.256	-0.785, 0.158 b

effect of location DMI $\beta2_{tropical}$	-0.062±0.277	-0.667, 0.441 b
effect of years since disturbance $\beta3_{boreal}$	-0.556±0.469	-1.48, 0.349 a
effect of years since disturbance $\beta3_{Mediterranean}$	1.08±0.691	-0.277, 2.53 a
effect of years since disturbance $\beta3_{temperate}$	0.263±0.243	-0.155, 0.775 a
effect of years since disturbance $\beta3_{tropical}$	**0.361±0.016**	**0.328, 0.394 a**
$\sigma\beta^2_{study\ random\ effects}$	0.923±0.312	0.490, 1.68
**Analysis of severity of disturbance**		
effect of disturbance severity $\gamma_{adult\ trees}$	**-0.567±0.040**	**-0.646, -0.488 a**
effect of disturbance severity $\gamma_{all\ strata}$	**0.223±0.036**	**0.161, 0.304 b**
effect of disturbance severity $\gamma_{seedlings}$	**0.659±0.039**	**0.581, 0.741 c**
$\sigma\gamma^2_{study\ random\ effects}$	0.880±0.186	0.583, 1.31
